# Co-localization and confinement of ecto-nucleotidases modulate extracellular adenosine nucleotide distributions

**DOI:** 10.1371/journal.pcbi.1007903

**Published:** 2020-06-25

**Authors:** Hadi Rahmaninejad, Tom Pace, Shashank Bhatt, Bin Sun, Peter Kekenes-Huskey

**Affiliations:** 1 Department of Physics and Astronomy, University of Kentucky, Lexington, Kentucky, United States of America; 2 Paul Laurence Dunbar High School, Lexington, Kentucky, United States of America; 3 Department of Chemistry, University of Kentucky, Lexington, Kentucky, United States of America; 4 Department of Cell & Molecular Physiology, Loyola University Chicago, Chicago, Illinois, United States of America; University of Houston, UNITED STATES

## Abstract

Nucleotides comprise small molecules that perform critical signaling roles in biological systems. Adenosine-based nucleotides, including adenosine tri-, di-, and mono-phosphate, are controlled through their rapid degradation by diphosphohydrolases and ecto-nucleotidases (NDAs). The interplay between nucleotide signaling and degradation is especially important in synapses formed between cells, which create signaling ‘nanodomains’. Within these ‘nanodomains’, charged nucleotides interact with densely-packed membranes and biomolecules. While the contributions of electrostatic and steric interactions within such nanodomains are known to shape diffusion-limited reaction rates, less is understood about how these factors control the kinetics of nucleotidase activity. To quantify these factors, we utilized reaction-diffusion numerical simulations of 1) adenosine triphosphate (ATP) hydrolysis into adenosine monophosphate (AMP) and 2) AMP into adenosine (Ado) via two representative nucleotidases, CD39 and CD73. We evaluate these sequentially-coupled reactions in nanodomain geometries representative of extracellular synapses, within which we localize the nucleotidases. With this model, we find that 1) nucleotidase confinement reduces reaction rates relative to an open (bulk) system, 2) the rates of AMP and ADO formation are accelerated by restricting the diffusion of substrates away from the enzymes, and 3) nucleotidase co-localization and the presence of complementary (positive) charges to ATP enhance reaction rates, though the impact of these contributions on nucleotide pools depends on the degree to which the membrane competes for substrates. As a result, these contributions integratively control the relative concentrations and distributions of ATP and its metabolites within the junctional space. Altogether, our studies suggest that CD39 and CD73 nucleotidase activity within junctional spaces can exploit their confinement and favorable electrostatic interactions to finely control nucleotide signaling.

## Author summary

Nucleotides are important signaling molecules. Their relative concentrations on the extracellular side of plasma membranes are tightly-controlled by nucleotidases. CD39 and CD73 are two such nucleotidases that sequentially convert the nucleotide adenosine tri-phosphate into adenosine, yet little is known about the efficiency of these enzymes in synapse-like junctions formed between neighboring cells. We therefore performed computer simulations of CD39- and CD73-catalyzed reactions in synapse-like junction geometries to understand how these enzymes work between neighboring cells relative to in vitro (cell-free) conditions. Our simulations reveal how properties like cell membrane charge and CD39/CD73 co-expression determine the relative balance of ATP and Ado along the cell exterior. Our approach and quantification of CD39 and CD73 activity at the cell membrane is expected to yield important insights into diverse nucleotidase-dependent phenomena, including tumor growth and regulation of immune responses.

## Introduction

Nucleotide signaling and regulation of cellular energy pools are reliant on the diffusion of small molecules over micrometer-scale distances [1]. Examples of processes reliant on nucleotides include signal transduction and regulation in smooth muscle [2], network motifs in transcriptional regulation networks [3], genomic regulatory networks [4], complexes of metabolic enzymes [5] and transmembrane ligand-gated channels [6, 7]. Of the latter, many nucleotide-gated channels and ATPases [8] reside within extracellular junctions formed between cells in close apposition [9], such as junctions between neurons and glia [10]. Scanning electron microscopy has revealed that many of these junctions are on the nanometer length-scale [11]. Within those junctional spaces, volumes can approach the femtoliter scale, within which we expect nucleotide free diffusion and apparent concentration of nucleotides will differ substantially from rates in bulk solutions. Nevertheless, how electrostatic interactions and confinement within junctions influence nucleotide diffusion and local concentrations have only been examined in limited detail.

At the cell surface, nucleotide distributions are controlled by phosphohydrolases and nucleotidases [12, 13]. These enzymes hydrolyze nucleotides including adenosine- and uracil-based molecules and thereby regulate the pool of nucleotides available for signaling and metabolism [14]. For example, it has been demonstrated that the nucleotide concentration can vary considerably at the cell surface on both the cytoplasmic and extracellular sides of the plasma membrane, as measured by the activity of ATPases and ATP-sensitive channels [15, 16]. Contributing to these variations in nucleotide concentrations is the activity of a sub-class of phosphohydrolases called NDAs, which are localized to the extracellular surfaces of cell membranes. There, NDAs rapidly and dynamically control nucleotide concentrations adjacent to extracellular and transmembrane proteins that catalyze or are gated by these molecules. Examples of such proteins include purinergic receptors, which are triggered by ATP and adenosine diphosphate (ADP) binding [14]. Many NDAs are relatively nonspecific in their affinities for adenosine-based substrates. However, some classes are selective for ATP and ADP, such as CD39a and CD39b, which catalyze these substrates into AMP [12, 17], or for AMP, such as the CD73 ecto-nucleotidase [12], which hydrolyzes AMP into Ado. These nucleotidases can be found in both freely-diffusing and membrane-bound forms [18]. Interestingly, when CD39 and CD73 are co-expressed, such as in T-cells [19] and B-cells [20], they catalyze the coupled, sequential and rapid [21] hydrolysis of ATP into adenosine and thereby influence nucleotide signaling within the extracellular space. However, precise characterizations of nucleotide diffusion-limited reaction kinetics catalyzed by nucleotidases within nanoscale junctional volumes are lacking. This limitation challenges quantification of nucleotide pools formed in extracellular compartments and their impact on nucleotide-dependent extracellular proteins governing diverse physiological functions.

A key foothold for quantifying extracellular nucleotide pools is to examine the coupling of CD39 and CD73 in their catalysis of ATP → AMP and AMP → Ado, respectively. This motif is typical of sequentially-controlled enzymatic processes that utilize two enzymes, whereby the first generates a reaction intermediate that is catalyzed by the second [5, 22]. For diffusion-limited reactions, the efficiency of sequentially-coupled reactions is strongly determined by the relative distance between enzymes, as well as the rates of substrate diffusion toward their reactive centers [23]. Further, sequential enzyme reactivity depends on the transfer efficiency of intermediates, which can be facilitated by molecular tunnels [24] or electrostatic channeling [23, 25]. An essential consideration is therefore how intrinsic rates of substrate diffusion in bulk solution are modulated by steric and long-range electrostatic interactions between substrates with their target enzymes and surrounding cellular environment [26, 27]. For instance, nucleotides are generally negatively-charged and are thus attracted to positively-charged nucleotide binding sites of typical NDAs [28]. Additionally, diffusion limitations stemming from densely packed media or impermeable membranes can confine substrates to narrow ‘microdomains’, within which substrate concentrations are vastly different from those in the bulk cytosol or extracellular medium [29]. Based on these considerations, it is plausible that nucleotidases confined to narrow compartments between cells will hydrolyze extant nucleotide pools with markedly different kinetics than those observed *in vitro*. In a recent and seminal study, Sandefur *et al* [30] modeled extracellular NDA activity in pulmonary epithelia, representing a first step toward describing coupled NDA enzyme activities. However, the reliance of the model on spatially-independent mathematical equations is insufficient to probe how long-range electrostatic interactions and confinement effects influence collective NDA activity.

In general, reaction kinetics in biological media are inherently difficult to study, given the breadth of influential factors including weak interactions of substrates with lipid membranes or proteins, restricted accessible volumes owing to crowding by nearby enzymes, proximity between enzymes involved in catalysis, and long-range electrostatic interactions. Since the systematic control of these factors in experiments is challenging [31], numerical models of molecular diffusion and reaction kinetics have been valuable in our understanding of catalysis in biological systems. At the coarsest resolution of such numerical models are representations of processes as networks of reactions and network motifs [22, 32–35]; although these coarse representations often do not account for kinetics or enzyme proximity, they have helped to establish bounds on the function of strongly-coupled reaction networks [36]. More sophisticated models accounting for enzyme size [37–40], charge [41, 42] and co-distribution [43] are based on ordinary and partial differential equation formalisms that implicitly capture these effects, including a recent study of nucleotide activity via ordinary differential equation (ODE) representations [44]. Recent ODE approaches that implicitly consider the distributions of finite-sized enzymes include a mean field theory from [45] and [46]. These models provided strong quantitative insights into the efficiency of catalytic processes [45] and limits on efficiency gains for sequentially-coupled enzymes [46], but only implicitly account for geometrical and physiochemical factors. Explicit consideration of those factors for coupled enzyme processes generally involve partial differential equations or particle-based solutions, which have afforded descriptions of how neighboring reactive enzymes [47–50], feedback inhibition, [45, 49], protein geometry and electrostatic interactions [23, 25, 51, 52] contribute to enzyme activity, but have not been applied to NDA-dependent processes. We therefore extend these approaches by modeling in spatial detail the concerted hydrolysis of nucleotides by CD39 and CD73 nucleotidases.

## Results

### Overview

**Fig 1:**
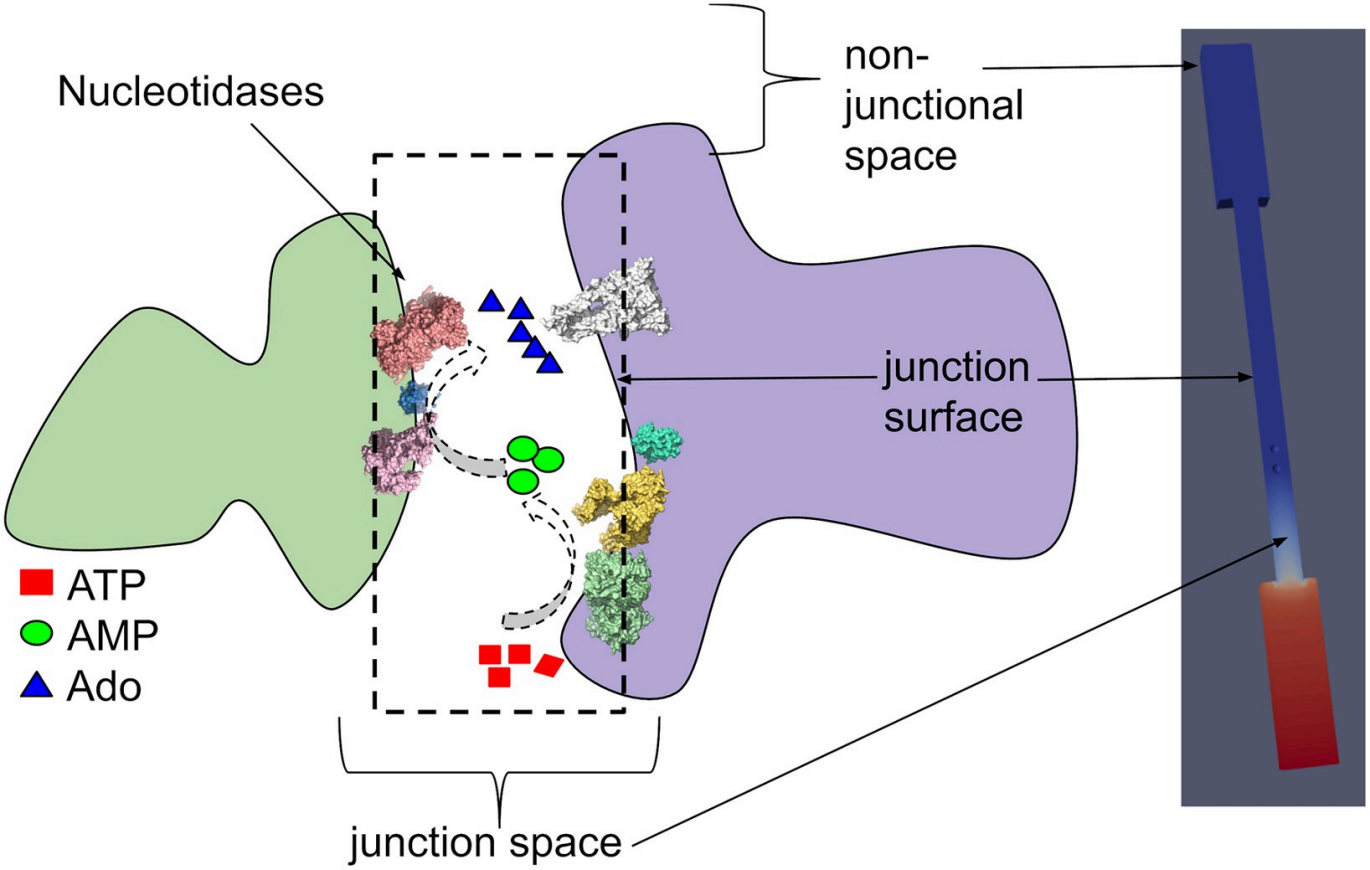
Systems studied in present work. Left) Schematic of a synapse-like junctional space formed between adjacent cells. Nucleotidases confined within the junctional space hydrolyze adenosine triphosphate (ATP) into adenosine monophosphate (AMP) and adenosine (Ado) via CD39 and CD73, respectively. Right) The schematic is emulated with a model junction geometry, for which the reservoirs correspond to the extracellular environment surrounding the junctional space. The spatial and electrostatic configurations of the mock synaptic junction influence the reactivity of confined nucleotidases CD39 and CD73, which in turn control the local concentration of nucleotide signals.

We used a computational model to quantify how the configuration of the CD39 and CD73 NDAs within closely apposed plasma membranes control sequential nucleotidase activity and local nucleotide pools. These enzymes catalyze the conversion of ATP directly to AMP, and AMP to Ado, respectively. This study was investigated in a model junctional geometry, for which the material porosity and surface composition could be controlled (Fig 1). The geometry of the model is further explained in Model geometry. Our approach used a finite element-based partial-differential equation model developed in [53], for which we introduced explicit enzymes [27] to quantify how conditions such as lipid charge, ionic strength, and localization to the membrane tune the efficiency of protein functions that utilize diffusing nucleotide substrates. We imposed an ATP gradient oriented parallel to the junction to emulate ATP diffusion from the surrounding environment toward the CD39 and CD73 enzymes. With this model, we examine how enzyme co-localization, ‘tethering’ the enzymes to the junction wall (to represent membrane-bound configurations), and charges on the enzyme and junction surfaces shape enzyme kinetics within the junction volume, as summarized in Theory. Our key findings are that NDA co-localization and their charge complementarity with substrates can offset reduced reaction rates from their confinement to nanoscale junctional volumes; moreover, tuning of junctional membrane surface properties, such as charge density, further improves nucleotidase reaction efficiency and modulates resulting nucleotide pools. These studies confirm that CD39 and CD73 sequential activity is strongly influenced by the configuration of their extracellular, junctional environment, and yield reaction kinetics that differ remarkably from bulk-phase in vitro comparisons.

### Effects of molecular junction confinement on enzymatic activity

Coupled enzyme reactions have been widely studied in a variety of contexts, including isolated globular enzymes and along surfaces. Here, we extend these approaches to examine nucleotide hydrolysis reaction kinetics for NDA enzymes embedded within nanoscale gaps between cells, which we emulated with junctions of varying widths (see Fig 1). These junctions are representative of small, well-contained extracellular volumes, such as junctions formed between adjacent cells or synapsing neurons. We vary the relative distance between enzymes to investigate how co-localization impacts reaction efficiency, as well as their distance to the junction membrane surface to simulate surface-tethered versus freely-floating enzymes. In such geometries, substrate access to the enzyme is restricted to a narrow volume, which is expected to decrease the diffusion-limited reaction rate. To quantify the dependence of enzyme reactivity on junction volume, we numerically solved reaction-diffusion models (Eq 12) for substrate species ATP, AMP and Ado subject to the boundary conditions defined in Theory. We defined the reaction efficiency as the ratio of the substrate Ado production rate coefficient, *k*_*prod*,*Ado*_ over the substrate ATP association rate coefficient, *k*_*on*,*ATP*_. We assume that the Ado production rate is equal to the association rate of AMP, hence the reaction efficiency highlights how the reactivity of AMP is shaped by the system configuration independent of the ATP reaction rate.

**Fig 2:**
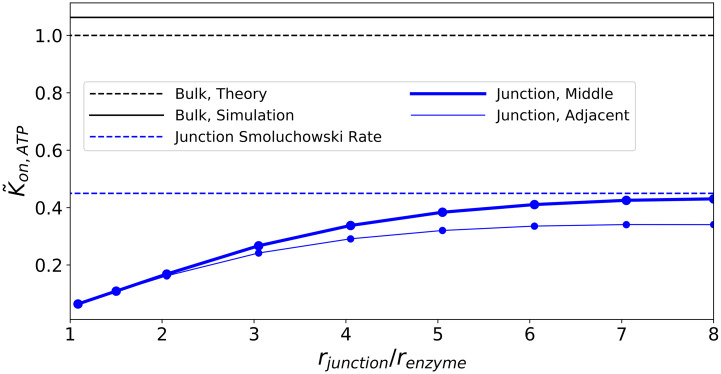
Effects of confinement and proximity. Predicted reaction rate coefficients for ATP to AMP at the CD39 enzyme, normalized to the analytical value from Eq 1. The bulk geometry is represented by a single CD39 enzyme in a junction of 16 nm radius with ATP = 1.0 mM on all boundaries (black). Results for different junction diameters are presented (in blue), including configurations where the enzyme is centered in the junction, or is adjacent to the junction wall.

We first validate our model against an analytical solution for the diffusion-limited reaction rate coefficient for a uniformly reactive sphere within a spherically symmetric, infinite domain. Here, the association rate coefficient, *k*_*on*_, for the reactive enzyme is given by [54, 55]:

$$\begin{array}{cc} k_{on} & =4\pi RD \end{array}$$ (1)

in which *R* is the radius of the enzyme, and *D* is the substrate diffusion coefficient. For the purpose of validation, we evaluate this rate at the sphere (2.0 nm radius) by assuming a uniform concentration for ATP (1.0 mM) at both the reservoir and junction (32.0 nm diameter). This configuration used a single CD39 enzyme in a cylindrical junction of radius of 16 nm and length of 80 nm. Thus, the enzyme concentration in this simulation is one particle in a junction volume of approximately 64,340 cubic nm, which is equivalent to roughly 0.03 mM. We hereafter refer to this as the ‘bulk’ configuration. Under these conditions, we numerically estimated a rate coefficient of *k*_*on*,*ATP*_ = 26.67 nm^3^ ns^−1^, which is within 6% of the analytical estimate of *k*_*smol*,*bulk*_ = 25.17 nm^3^ ns^−1^. This serves as our reference for the normalized rates presented in Fig 2. The minor discrepancy can be attributed to the nonspherical domain used for the numerical simulation, whereas a radially-symmetric domain is assumed in Eq 1.

To validate model predictions within the junction geometry, we define boundary conditions that are imposed at a finite radial distance, *αR*. Solving the Fickian diffusion equation analytically subject to a finite radial distance for the boundary condition yields the expression

$$k_{on}=4\pi RD\frac{\alpha }{\alpha -1}$$ (2)

from Eq 1. To validate our model for enzymes confined with the junction, we conducted a simulation with similar parameters to the bulk conditions described above, except that the boundary conditions along the junction were changed to reflecting boundary conditions (zero flux across the outer surface of the junction). Concentration data extracted from this simulation were assessed to find the radial distance at which the concentration field was no longer dominated by the presence of the CD39 enzyme, which was approximately at 3*R* (*α* = 3), based on visual inspection of the concentration values. At this distance, the average concentration of ATP was observed to be approximately 0.3 mM. Computing *k*_*on*,*ATP*_ from this simulation led to a result of *k*_*on*,*ATP*_ = 10.83nm^3^ ns^−1^. This value is within 5.0% of the result from using *α* = 3 in Eq 2, *k*_*smol*,*local*_ = 11.33 nm^3^ ns^−1^. These approaches (see also S1 Text) confirm the reliability of the computational model for reproducing analytic results for diffusion-limited association reactions.

Using our validated model we investigated how the reaction of substrate ATP on CD39 is influenced by confinement within the nanoscale junction. These simulations were conducted assuming a constant concentration gradient along the dominant axis of the channel. In Fig 2 we present normalized association rates for ATP with enzyme CD39, ${\tilde{k}}_{on}\equiv k_{on,ATP}/k_{on,Bulk}$, subject to a constant enzyme radius (*r*_*E*_ = 2.0 nm) and varied junction radii (*r*_*j*_ ≈ 2− 16 nm). Confinement of a single enzyme to the junction reduced the reaction rate coefficient by roughly 60-80% relative to the corresponding rate in bulk (${\tilde{k}}_{on}=1$)(Fig 2). Some of this decline is due to imposing an ATP concentration gradient across the junction, whereas the bulk cases utilized a constant ATP value at its outer boundary. This rate reduction can be qualitatively rationalized by the concentration profiles manifest in the channel (see Fig A in S1 Figures). The concentration profile decreases from [ATP] = 6.0 × 10^-4^ nm^−3^ at the right-hand side reservoir (Γ_*R*_) and approaches zero at the left-hand side reservoir (Γ_*L*_). Further, as the junction radius decreases from from *r*_*j*_ ≈ 8*r*_*E*_ to *r*_*j*_ ≈ *r*_*E*_, the concentration of ATP within the junction decreased even more relative to the source reservoir. Both factors reduce the substrate concentration at the enzyme surface, which culminates in a reduced *k*_*on*,*ATP*_.

We additionally varied the proximity of the enzyme to the junction surface. This served as a proxy for probing the reactivity of enzymes that are essentially freely floating within the junction interior versus immobilized to the junction surface. The reactivity of CD39 was additionally reduced, albeit negligibly, as CD39 was localized to the junction surface. This is most likely due to the reduction in the substrate-accessible volume around the enzyme. This effect can also be rationalized by noting the similarity between the time-independent diffusion equation and the Laplace equation commonly used in electrostatics (see Eq 15 with *κ* = 0 and rationale in S1 Text). Altogether, these results demonstrate that restricting the diffusion of ATP within the junction and to a slightly greater extent, near the junction wall, suppress *k*_*on*,*ATP*_ relative to the bulk.

**Fig 3:**
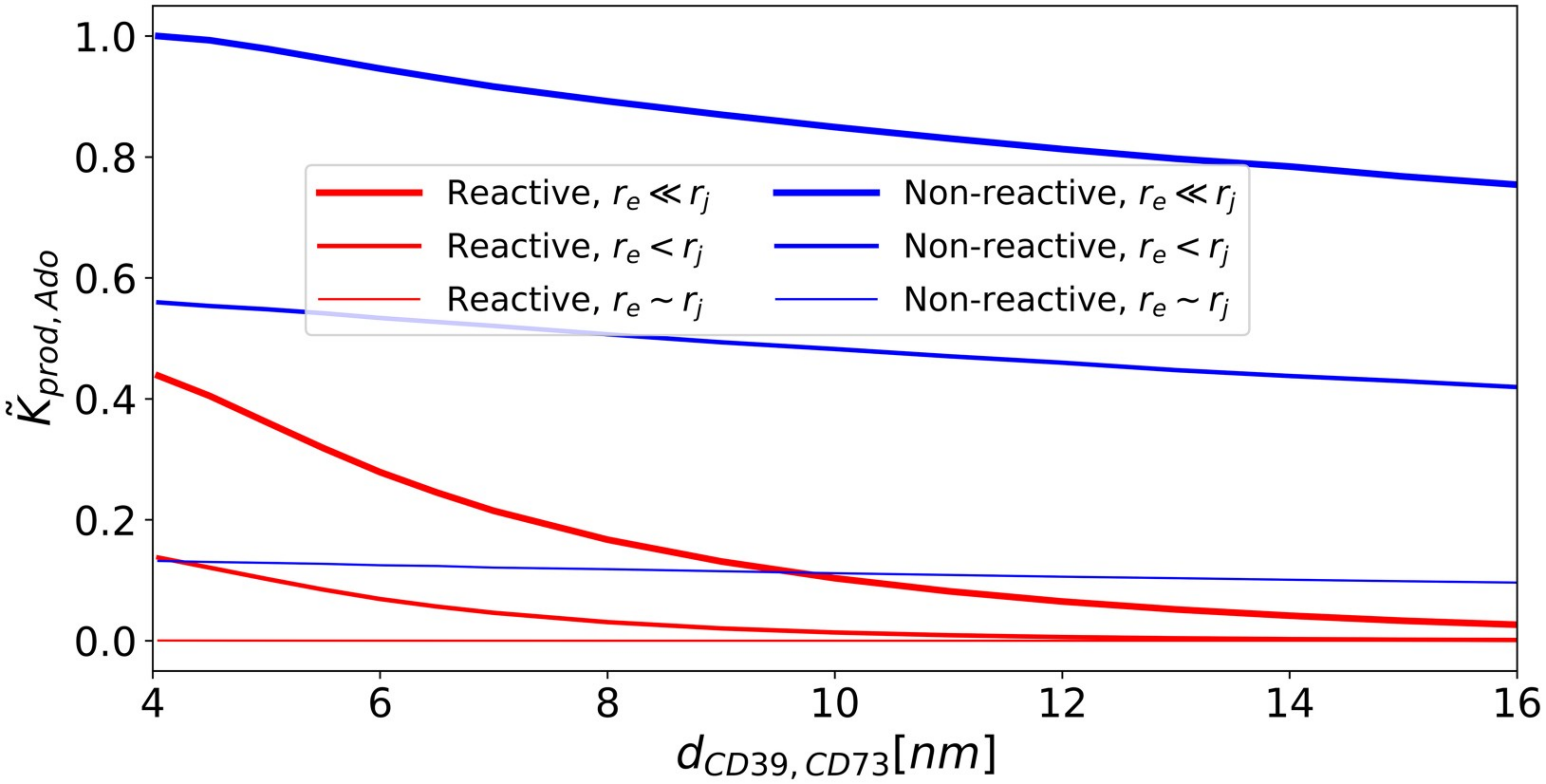
Effects of nucleotidase confinement and co-localization on on adenosine (Ado) production rates. Normalized reaction rate coefficient, *k*_*prod*,*Ado*_, for CD73 with different junction sizes and CD39/CD73 proximities. ${\tilde{k}}_{prod,Ado}$ is normalized with respect to the maximal *k*_*prod*,*Ado*_ value, which is found under conditions of minimal enzyme separation distance and maximal junction radius. Red lines are for absorbing boundary conditions to emulate conditions where the membrane competes for the AMP intermediate. Blue lines are for reflective boundary conditions to represent membranes that are non-reactive to the substrate. The line thickness is proportional to the radius of the junction.

Reduction of *k*_*on*,*ATP*_ through confinement of enzymes is expected to subsequently suppress production rates for AMP and Ado. In practice, to compete with this reduction, enzymes are often co-localized to tune production rates of desired chemical products [43, 45, 56]. We therefore introduced a second enzyme, CD73, into the junction and simulated the steady state reactions *ATP* → *AMP* at CD39 and *AMP* → *Ado* at CD73. In Fig 3 we first report Ado production rate coefficients, *k*_*prod*,*Ado*_, as a function of enzyme separation and for junction surfaces that are non-reactive (reflective) or reactive (absorbing) for the AMP intermediate. These values are normalized with respect to the *k*_*prod*,*Ado*_ value obtained for *r*_*j*_ ≫ *r*_*E*_ and minimal enzyme separation (*d*_*CD*39,*CD*73_ ≈ 4nm). The normalized *k*_*prod*,*Ado*_ rate coefficients are negligibly impacted by decreasing enzyme separation in the presence of boundaries that are nonreactive with AMP. This indicates that enzyme colocalization has negligible impact on *k*_*prod*,*Ado*_ under non-reactive boundary conditions. In contrast, we use absorbing conditions to represent scenarios in which enzymes auxiliary to NDAs can deplete nucleotide intermediates. Here we observe a kinetic advantage to enzyme colocalization that is demonstrated by the increased rates with reduced *d*_*CD*39,*CD*73_ in Fig 3, which are further accentuated as the junction size increases. Hence, as the domain approaches the bulk-like system where AMP can escape from the reaction complex, the advantage of enzyme proximity becomes apparent and is consistent with recent studies of enzyme co-localization [23, 45, 46]. In summary, the nature of the intermediate (AMP) interactions with the surface appear to determine the relative advantage of enzyme colocalization in closed, nanoscale domains.

**Fig 4:**
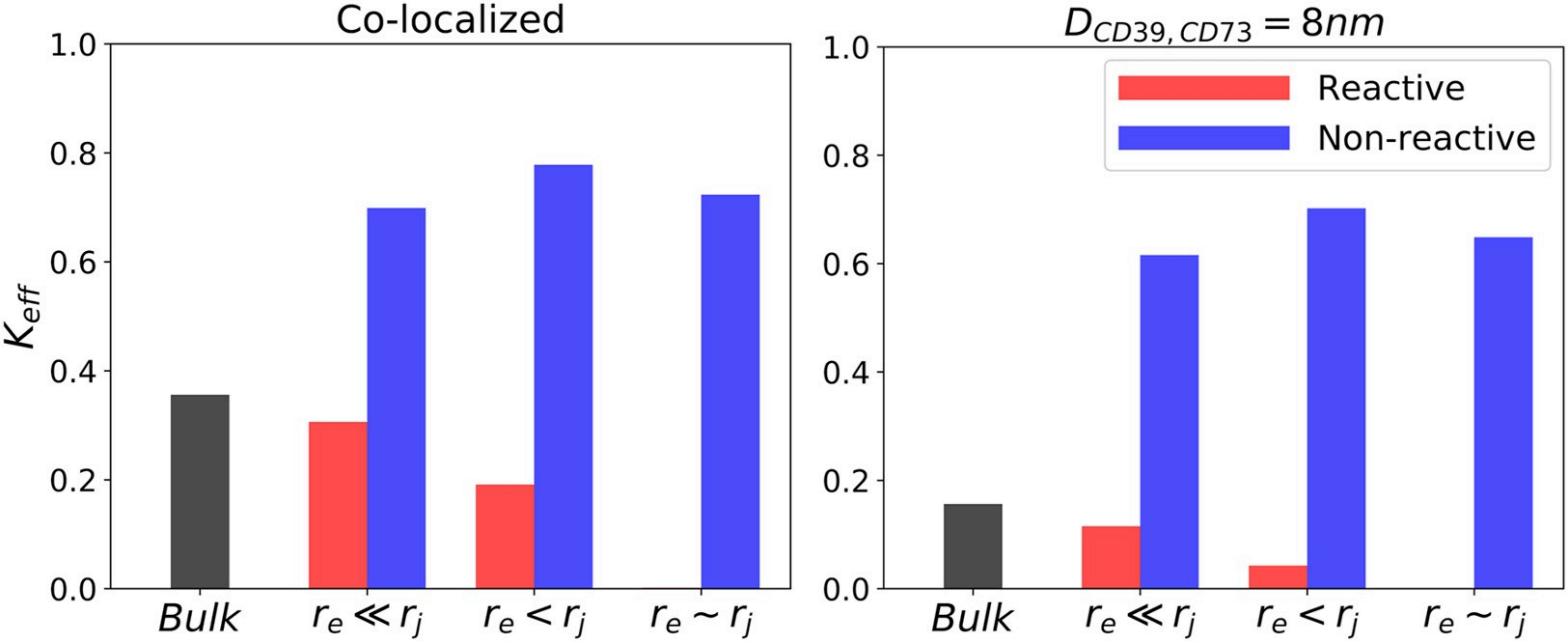
Effects of nucleotidase confinement and co-localization on Ado production efficiency. Efficiency of reactivity of CD73 with respect to CD39 defined as *k*_*eff*_ ≡ *k*_*prod*,*Ado*_/*k*_*on*,*ATP*_. Left panel: co-localized enzymes. Right panel: enzymes separated by 8 nm.

To delineate the effects of junction confinement and enzyme colocalization on *k*_*on*,*AMP*_ and *k*_*prod*,*Ado*_ normalized for *k*_*on*,*ATP*_, we report in Fig 4 the Ado production efficiency, *k*_*eff*_, which we define as *k*_*eff*_ ≡ *k*_*prod*,*Ado*_/*k*_*on*,*ATP*_. The efficiencies reported for co-localized enzymes (left panel) are consistently higher than those for separated enzymes (right panel), with the largest increases demonstrated for junction boundaries reactive to AMP (red) and bulk (black) configurations. Hence, under circumstances that permit intermediates to diffuse away from or compete with the reactive centers, there is a clear advantage to colocalization, akin to findings in [46]. However, in the absence of substrate interactions with the junction surface, confinement leads to higher overall efficiencies, with little dependence on enzyme proximity.

**Fig 5:**
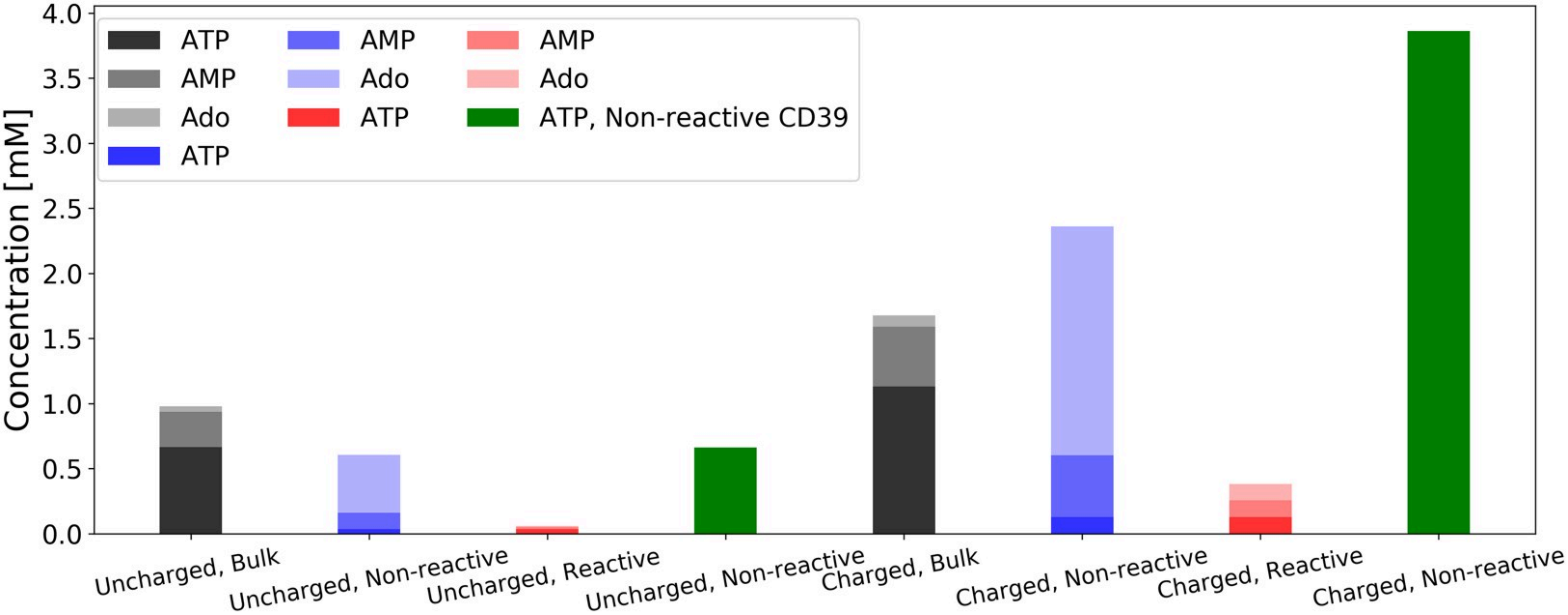
ATP, AMP and Ado concentrations at midpoint between enzymes. CD39 and CD73 are separated by a distance of 10.0 nm. In the uncharged cases, all surfaces are electrically neutral. In the charged cases, surface potentials are applied as described in Effects of surface charge on reaction rate coefficient. Bulk conditions (black and gray) are as described in Effects of molecular junction confinement on enzymatic activity. In non-reactive cases (blue) the junction surface reflects nucleotides, while in the reactive cases (red) the junction surface absorbs AMP. Cases are also shown (green) where the CD39 enzyme is inactive, resulting in no production of AMP or Ado.

In Fig 5 we summarize the relative concentrations of ATP and its derivatives maintained in the junction by sequentially-coupled NDAs. We present results for nucleotide concentrations at the mid-point between the CD39 and CD73 enzymes separated by 10 nm, with a 1 mM ATP concentration imposed at the reservoir boundary. These data (uncharged bulk, uncharged nonreactive, and uncharged reactive membrane) indicate that confinement generally reduces the total nucleotide concentration relative to bulk. However, confinement yields a higher ratio of Ado versus ATP compared to the bulk condition.

### Effects of surface charge on reaction rate coefficient

In the previous section we demonstrated that confinement of CD39 and CD73 to nano-scale extracellular compartments suppresses the overall reaction rate coefficient of uncharged substrates. Further, co-localization of the reactive centers mitigated this reduction to a modest extent. However, adenosine substrates are generally charged, with ATP having the most negative charge and Ado the least. Hence, their concentrations and diffusion rates are expected to be sensitive to the charge configuration of their binding partners and the surrounding lipid bilayer environment. It is well-known, for instance, that many enzymes have evolved to exploit electrostatic interactions to accelerate substrate binding [41]. Further, there is strong evidence that local ionic concentration near charged membranes yield concentrations of charged species like Ca^2+^ and Na^+^ that deviate significantly from the bulk [57, 58]. We therefore expanded the approach in the previous section to consider competing or complementary effects of electrostatic interactions in coupled NDA activity.

We first validate our model with electrostatic interactions using the Smoluchowski electro-diffusion equation (see Eq 8), for which the electrostatic potential was modeled using the linearized Poisson-Boltzmann equation (see Eq 15). We first validate our implementation under dilute solvent conditions (*κ* → 0) and assume that the junction and CD73 are uncharged. The association rate in this configuration for a substrate with charge *q*_*A*_ with a spherically-symmetric enzyme, *E*, of radius, *r*_*E*_ and charge *q*_*E*_ can be analytically determined [23]:

$$\begin{array}{c} k=4\pi D(\frac{Q_{s}\text{exp}\left(Q_{s}/r_{D}\right)}{\text{exp}\left(Q_{s}/r_{E}\right)-\text{exp}\left(Q_{s}/r_{D}\right)}) \end{array}$$ (3)

with *Q*_*S*_ ≡ *q*_*A*_ *q*_*E*_λ_*B*_, where *r*_*D*_ is the radius of the domain within which the reaction is confined and λ_*B*_ is the Bjerrum length. Accordingly, we demonstrate in Fig B in S1 Figures that for *κ* → 0 that the *k*_*on*,*ATP*_ rates for *q*_*A*_ = −1 and *q*_*E*_ ∈ [0, 3] approach the analytical estimate within 15%, and thus reasonably validate the electrostatic model. We attribute the discrepancy in part due to the non-spherical system geometry, whereas an exact sphere is assumed in Eq 3.

Using the validated electro-diffusion model, we examined changes in CD39 reactivity by confinement within an electrically-neutral junction, subject to electrostatic interactions between a negatively charged substrate ATP, positively charged CD39, and CD73 with varying charges. We assumed surface potentials of ±25.6 mV (equivalent to $1\frac{kT}{e}$) for the enzymes which is on the same order of the *ζ* potentials measured for proteinaceous solutions by Salgn *et al* [59]. Further, although adenosine metabolite charges vary from -4 to 0, ATP is commonly chelated by Mg^2+^ [60], therefore we used charges of -2, -1 and 0 for ATP, AMP and Ado, respectively, to exemplify effects on reactivity.

We next imposed a negative electric potential on the junction surface and present the resulting reaction rate coefficients (red in Fig 7). We chose surface charge densities consistent with biological membranes reported by surface conductivity microscopy such as DPTAP = 15.1 mC m^−2^, DPPE = 5.3 mC m^−2^ and DPPG = −44.0 mC m^−2^ for positively charged, zwitterionic and negative charged lipid bilayers, respectively [61]. An equivalent electrical potential for these charge densities can be obtained from the Graham equation (Eq 16, in Theory).

In Fig 5, we first report nucleotide concentrations for non-reactive CD39 (green) to indicate how the enzyme and surface charges influence the distribution of ATP relative to the nucleotidase centers, in the absence of enzyme activity. This is done to delineate contributions of the membrane charge to the local concentration before enzyme kinetics are considered. Namely, we observe a moderate reduction in ATP in the uncharged system relative to the concentration imposed at the reservoir boundary. As a significant attractive charge at the membrane is imposed, the local ATP concentration increases several fold. Comparison of the charged and uncharged nonreactive conditions is suggestive of how electrostatic interactions localize substrate toward the enzymes and thereby improve reaction efficiency.

**Fig 6:**
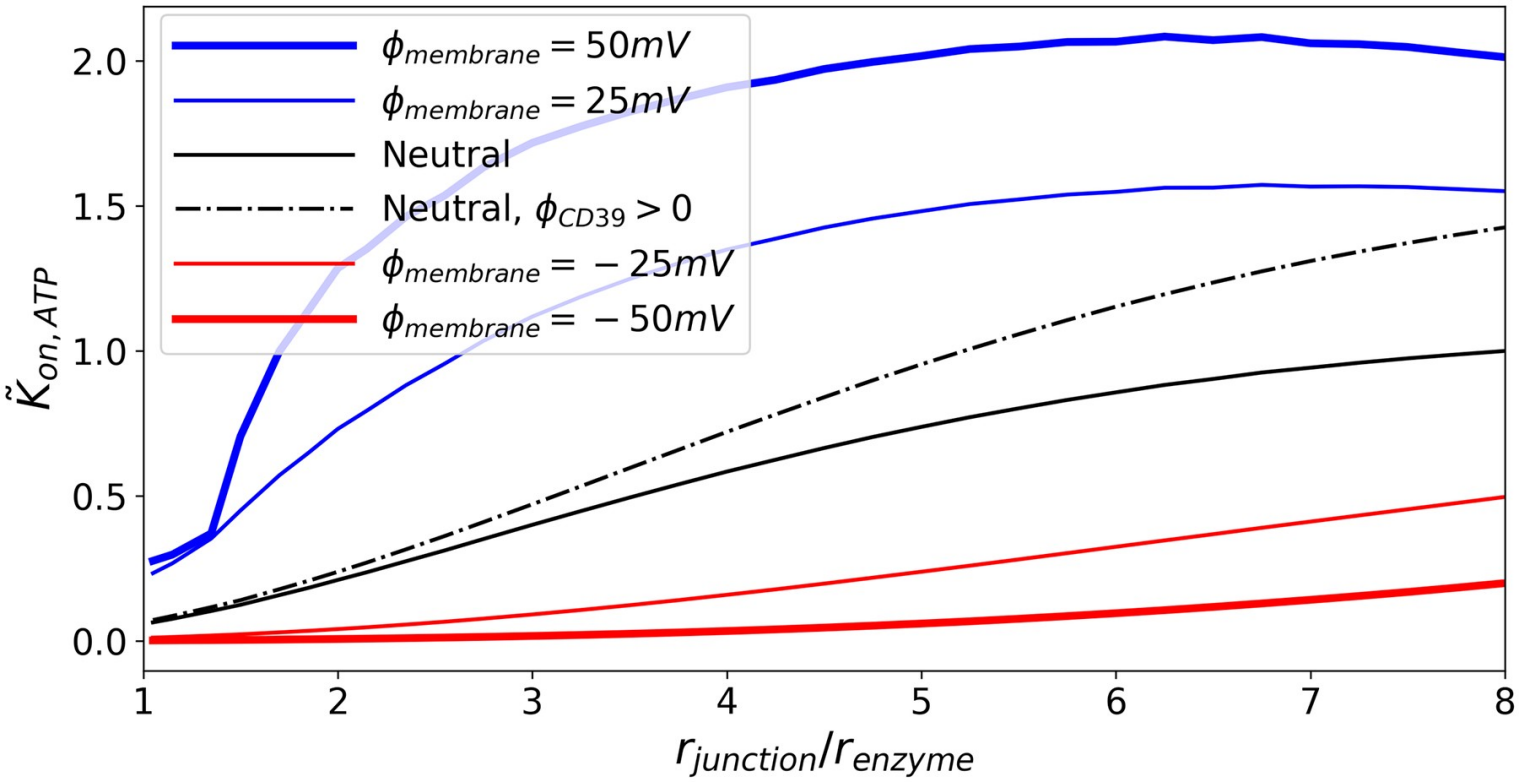
Effects of junction membrane electrical potential on CD39 reactivity. *k*_*on*,*ATP*_ is shown for various junction radii. Black curves represent cases where the junction membrane surface is electrically neutral. Blue curves represent cases where the junction membrane surface has an electric potential that attracts ATP. Red curves represent cases where the junction membrane surface has an electric potential that repels ATP. Φ_*CD*39_ = 0, except where noted.

Under conditions of charged proteins and substrates with neutral membranes, *k*_*on*,*ATP*_ decreases as the ratio of the radius of the junction to the enzyme decreases, consistent with observations for the neutral system (see Fig 6). Importantly, we note that *k*_*on*,*ATP*_ for the charged enzyme does assume a higher rate coefficient than the neutral system; in this capacity, the electrostatic interactions 1) counterbalance the reduction in reaction rate coefficients due to confinement and 2) achieve rapid association through nucleotide/NDA charge complementarity.

We next examine effects of the junction electric potential, Φ_*junction*_, on reaction kinetics, assuming CD73 is uncharged. In Fig 6 we demonstrate that in general *k*_*on*,*ATP*_ monotonically decreases with reducing junction size regardless of the membrane charge. In the event that the junction interactions with substrate ATP are repulsive (Φ_*junction*_ < 0), the reaction rate coefficient decreases at a faster rate. However, in certain regimes the charge complementarity of the junction surface was found to greatly accelerate *k*_*on*,*ATP*_ relative to the neutral junction, whereas a repulsive junction (Φ_*junction*_ < 0, red) attenuated *k*_*on*,*ATP*_ by more than 30% for most of the junction size range. We attribute the enhanced reaction rate coefficient for the positively charged membrane to the elevated concentration adjacent to the membrane relative to that of the uncharged membrane. Namely, the complementary charged junction surface drew ATP into the junction interior and thereby facilitated the reaction on CD39. Hence, the charge of the junction surface stemming from different phospholipid compositions can strongly influence *k*_*on*,*ATP*_, and in turn ultimately controls AMP production.

Interestingly, attractive junction/ATP interactions initially accelerate *k*_*on*,*ATP*_ as the junction diameter is reduced, whereafter the rate declines. We find the maximal acceleration is achieved when the junction size is roughly seven-fold higher than the enzyme radius. This maximum is dependent on the wall potential amplitude. Namely, as the attractive wall potential amplitude increases, the maxima shift to smaller junction/enzyme size ratios. In previous studies [53, 62, 63], it has been demonstrated that weakly attractive interactions with junction boundaries can enhance diffusion and ion conductivities, which is consistent with the initial increase in *k*_*on*,*ATP*_ in our model. However, this acceleration in diffusion due to attractive interactions is eventually outweighed by the hindrance of diffusion as the junction is narrowed. Additionally, although diffusion is likely accelerated, the amount of substrate able to interact with the target is reduced, as we observed for the uncharged cases. Hence, attractive junction potentials serve to co-localize substrates near the junction wall and therefore offset the reduced reaction volume that would otherwise decrease the overall reaction rate coefficient (see Fig C in S1 Figures, especially for high ionic strength).

Similarly, we initially anticipated that anchoring the protein to the cell membrane (a condition approximated by placing the protein adjacent to the membrane surface) would improve the reactivity relative to the junction center. While we observed that *k*_*on*,*ATP*_ can be amplified when the enzymes are tethered to the junction surface under specific conditions, namely wide junctions and strong attraction, the advantage is generally minor and thus of limited consequence to NDAs (Fig C in S1 Figures). This appears to be consequence of reduced ability for the substrate to access the enzyme when adjacent to the junction membrane, which counterbalances the increased the concentration of ATP near the surface due to attractive electrostatic interactions.

**Fig 7:**
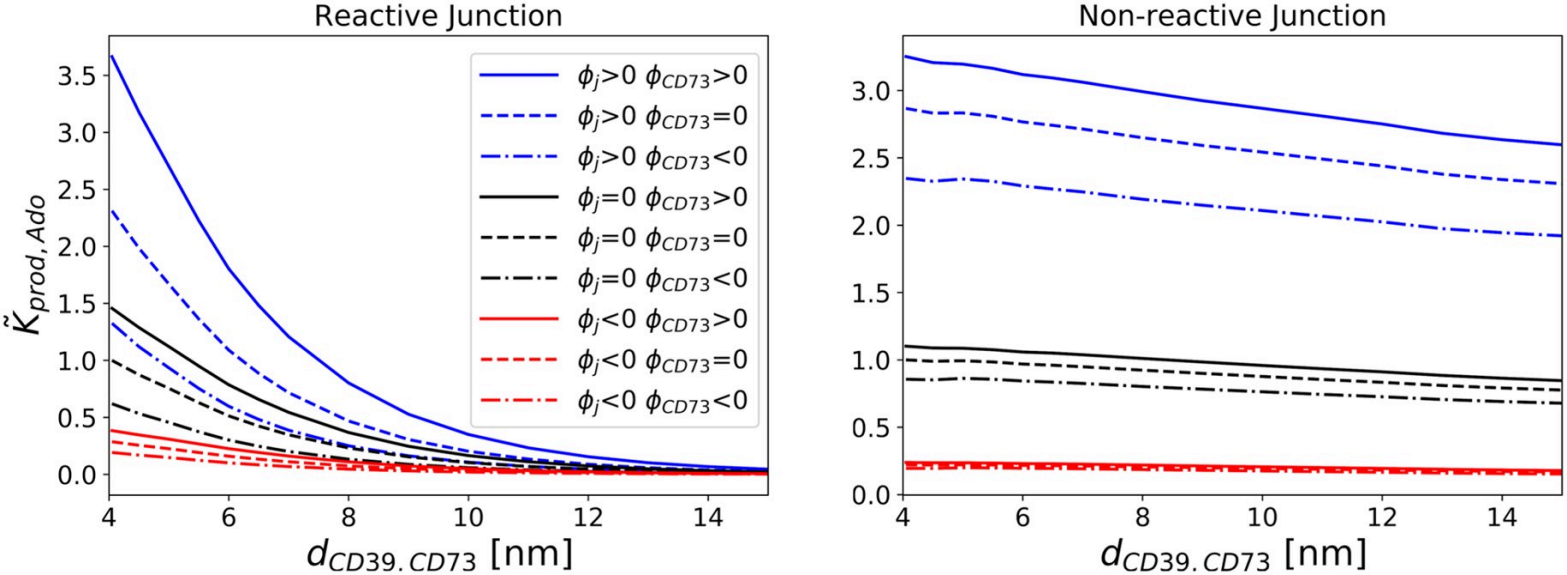
Effects of CD73 and membrane electric potential on CD73 reactivity. *z*_*ATP*_ = -2, *z*_*AMP*_ = -1, *z*_*Ado*_ = 0 and Ω_*CD*39_ > 0. Reaction rate coefficients for production of Ado as a function of distance between enzymes. The values are normalized to the *k*_*prod*,*Ado*_ value found for the minimal enzyme separation distance for each boundary condition under the neutral case. Left:reactive; right:Non-reactive to AMP.

Electrostatic enhancement of *k*_*on*,*ATP*_ is generally expected to promote *k*_*on*,*AMP*_ and *k*_*prod*,*Ado*_. Hence, we examined the extents to which the intermediate species’ charge and enzyme proximity influence *k*_*prod*,*Ado*_ (see Fig 7) assuming a negatively-charged AMP intermediate (*z*_*AMP*_ = -1). Consistent with findings from the neutral system and our previous studies of sequential enzyme channeling [23], we find that *k*_*prod*,*Ado*_ increases as the enzymes are brought into close proximity (${\tilde{d}}_{CD39,CD73}\to 0$). As observed in the preceding section, the absorbing junction boundaries show the greatest sensitivity to enzyme distance, with favorable AMP /CD73 electrostatic interactions for Ω_*CD*73_ > 0 yielding faster *k*_*prod*,*Ado*_ reaction rate coefficients relative to neutral CD73. Conversely, slower rates for negatively-charged CD73 were observed. The enhancement in the former case reflects the electrostatic attraction of substrate AMP toward the enzymes, which prevents it from diffusing away from the enzymes. This behavior was also reported in [23] for an unconfined (bulk) system, for which reaction rates were maximized when charged enzymes were co-localized. When CD73 was negatively charged, AMP was repelled from the enzyme, while being attracted to CD39, culminating in a substantial reduction in its reaction rate. Hence, the enzymes’ charge complementarity with their respective substrates, as well as the enzymes’ co-localization, together enhance the overall reaction rate coefficient relative to uncharged systems, consistent with [23, 45, 46]. Importantly, electrostatic enhancement effectively counterbalances the rate reduction due to confinement of the enzymes to the narrow junctional space. We discuss in S1 Text relationships between *k*_*prod*,*Ado*_ and the AMP/membrane interaction strength, as well as concommitant effects on the reaction efficiency *k*_*eff*_.

Overall, the trends observed for the relative nucleotide concentrations arising under bulk, non-reactive and reactive conditions for the charged systems shown in Fig 5 were consistent with the uncharged configurations. Importantly, we note that the ATP concentration is considerably higher for the bulk system when CD39 assumes a positive charge relative to the uncharged system, as the enzyme attracts its substrate. As a result, AMP and Ado concentrations were proportionally higher for both the reactive and nonreactive cases. In the confined systems that featured charged membrane and enzymes, ATP was depleted relative to the uncharged configuration as the membrane potential attracted the substrate away from the enzymes. Nonetheless, [AMP] and [Ado] were larger for the charged cases relative to their uncharged equivalents.

In Fig 5, comparing the respective nonreactive CD39 configurations (green) to their reactive CD39 equivalents (blue) demonstrates how reactivity of the enzymes modulates the steady state substrate concentrations. In the uncharged case, the total concentration of ATP and its derivatives is unchanged when the enzymes are activated. In contrast, the charged system presents a reduction in the total concentration of all nucleotide species; this is in part due to the charged membrane redistributing the differentially-charged substrates away from the reactive centers. Comparing the cases with non-reactive junction membranes (blue) to the bulk conditions (black) illustrates the effects of enzyme confinement. The total concentration of ATP and its derivatives within the junction assuming a nonreactive membrane is significantly reduced relative to the bulk case which stems from the junction restricting the diffusion of ATP toward the enzymes. For the reactive membrane configuration whereby AMP is consumed along the surface (red), the total nucleotide pool was greatly reduced, owing to dramatic reductions in the AMP and Ado pools. We anticipate that the more likely scenario of a spatially-heterogeneous reactive membrane or one that only consumes a fraction of the local AMP pool, will yield nucleotide distributions intermediate to the non-reactive and reactive configurations shown here.

**Fig 8:**
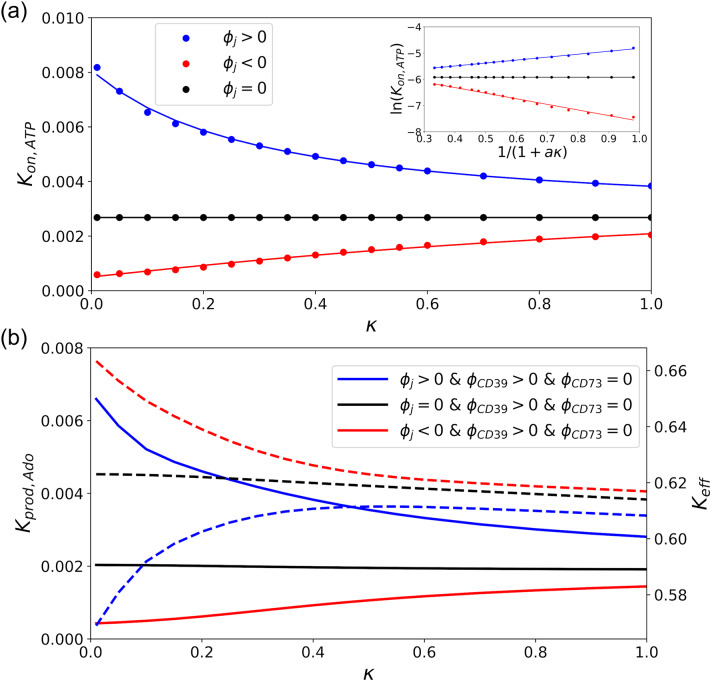
Effect of Debye Length on reactivity of the sequential enzymes. a)Reaction rate of CD39, *k*_*on*,*ATP*_, as a function of Debye length parameter (*κ*) which is proportional to the square root of ionic strength. The circle shows simulation data and the solid lines are obtained by fitting to Eq 4. The subplot shows the linear relationship between ln(*k*_*on*_) and (1 + *aκ*)^−1^. Linear regression of Eq 4 yields the interaction strength U/RT and ln *k*_*on*,*I* → ∞_ (see Table 1). b)Production and effective reaction rates versus *κ*. The solid lines correspond to the left axis (*k*_*prod*,*Ado*_) and the dashed to the right axis (*k*_*eff*_).

Lastly, we examined how the predicted reaction rates were influenced by electrostatic screening due to common electrolytes, such as KCl. To model these contributions, we solved the linearized Poisson-Boltzmann equation, assuming Debye lengths approaching 1 nm. This Debye length signifies that electrostatic interactions are significantly screened within the junction, which will suppress the attractive interactions driving the rapid reaction rates modeled in the previous section. To assess these effects on *k*_*on*,*ATP*_, we compare rates as a function of (1 + *aκ*), where *a* is the enzyme radius and *κ* is the inverse Debye length (see Fig 8). This functional form is motivated [64–66] by the relationship

$$\text{ln}\;k_{a}=\text{ln}\;k_{I\to \infty }-\frac{U}{RT}[\frac{1}{1+a\kappa }]$$ (4)

where U is the electrostatic interaction between the substrate and enzyme. This latter term stems from the Debye-Hückel treatment of electrolyte solutions.

In Fig 8(a), we demonstrate how ln[*k*_*on*,*A*_] scales with respect to (1 + *κa*)^−1^, assuming either attractive, repulsive, or inert substrate interactions with the junctional membrane. For attractive interactions, the maximum rate enhancement relative to an electrically-neutral reference system is found under dilute conditions signified by *κ* → 0 or equivalently, (1 + *κa*)^−1^ → 1. At high ionic strength (*κ* → ∞), the rates approach those of the neutral system. In Table 1, we demonstrate that our data are well described by Eq 4, as linear regression correlation coefficients were greater than 0.98. This fit allowed us to determine the effective interaction strengths, *U*/*RT*, for the repulsive, neutral and attractive cases as 2.1, 0.0 and -1.1, respectively. Accounting for screening led to a substantial reduction for *k*_*on*,*ATP*_ relative to the electrolyte-free conditions assumed in the previous section.

**Table 1:** Reaction rate at high ionic strength, interaction strength, and linear regression correlation coefficients by fitting of simulation data to Eq 4 in Fig 8.

*ϕ*	log *κ*_*I*→∞_	*U*/*RT*	*R*^2^
*ϕ* < 0	-5.5	2.1	0.988
*ϕ* = 0	-5.9	0.0	N/A
*ϕ* > 0	-5.9	-1.1	0.997

Since ATP reaction rates were suppressed by electrolyte screening, we also present data for the effects of enzyme co-localization and junction proximity on *k*_*prod*,*Ado*_ and *k*_*eff*_, subject to 150 mM KCl. As anticipated, *k*_*prod*,*Ado*_ generally scales proportionally to *k*_*on*,*ATP*_ as a result of ATP/junction interactions largely setting the overall reaction rate coefficient relative to the intermediate (see Fig 8b, left y-axis). However, interestingly, the trend was reversed for *k*_*eff*_ (see right y-axis), that is, repulsive AMP/membrane interactions led to more efficient *k*_*eff*_ values. This behavior can be attributed to the negatively-charged membrane repelling AMP toward CD79. Although the membrane/AMP repulsion improved reaction efficiency, the effect on *k*_*prod*,*Ado*_ was small relative to the contributions of ATP/membrane interactions. We additionally found that the trends in *k*_*eff*_ and *k*_*prod*,*Ado*_ relative to enzyme co-localization and junction proximity did not significantly deviate from those reported in electrolyte-free conditions and are therefore not reported here. Overall, while physiological ionic strength conditions modestly impact reaction kinetics relative to dilute conditions, the changes are fairly insignificant and also insensitive to moderate changes in ionic strength.

## Discussion

### Summary

In this study, we probed how nucleotide signals are controlled by ecto-nucleotidase (NDA) enzymes within narrow junctions formed between adjacent cells, similar to synaptic junctions between neurons. Here we extended numerical solutions of coupled nucleotidases that were recently studied [30] to include spatial and electrostatic factors known to influence enzyme kinetics. Our simulations were performed under physiological conditions that included confinement to narrow junctions bordered by cell plasma membrane and long-range, ionic strength-mediated electrostatic interactions. Our key findings were

- that independent of NDA activity, nucleotide distributions within confined extracellular junctions can significantly differ relative to open, bulk-like configurations
- that NDA steady-state reaction rates are generally smaller when localized to junctions, but the efficiency of generating nucleotide products like Ado can be increased by co-localizing coupled enzymes, and
- that these reaction rates can be substantially accelerated when NDA and plasma membrane adopt charges complementary to reacting substrates, especially when the membrane attracts the relevant substrate.

Adenosine nucleotides encompass a set of small, polar molecules that are critical for cellular signaling and metabolism [14]. These nucleotides are generated or regulated by diverse processes, including secretion from neighboring cells in tissue [67], as products of membrane-bound F1-F0 ATP synthases [68], transport via ectopic adenine nucleotide translocases [69] or hydrolyzed by by ecto-nucleotidase (NDA). For many cellular systems, these processes occur within femtoliter-scale [11, 70] regions between neighboring cells, such as those characteristic of neuronal synapses [71]. Here, co-localization of NDAs including CD39 with purinergic receptors within caveolae [72] or with extracellular ATP release sites on astrocytes [73] can give rise to ‘compartmented’ nucleotide pools [74] that can strongly influence nucleotide-dependent signaling. The thermodynamics and kinetics of molecular signaling in such compartments can differ considerably from analogous processes in bulk solutions or in vitro. Myriad factors contribute to these differences, including smaller compartment volumes that strongly modify substrate concentration gradients [29, 75], the presence of ‘crowders’ comprising other small molecules, protein or nucleic acid that generally impede diffusion [47, 76–78], as well as rate enhancements typically exhibited for closely-apposed enzymes [41, 46, 49, 52, 79] or those adopting electrostatic fields complementary to a reacting species [37]. The relative contribution of these factors to coupled NDA activity in a given multi-cellular domain has not been examined previously and could provide key details on the relative distribution of nucleotides adjacent to the plasma membranes. The specific balance of nucleotide concentrations is expected to determine the extent of activation for membrane bound, nucleotide receptors, ATPases and translocases, which shape diverse cellular processes, including migration [80] and cytokine release [81].

To orient our results, we note that increasing *k*_*on*,*ATP*_ rates (reaction of ATP at CD39) result in AMP accumulation, while large *k*_*on*,*AMP*_ and *k*_*prod*,*Ado*_ correspond to high rates of AMP consumption and Ado production at CD73. Since the latter rates are generally proportional to *k*_*on*,*ATP*_, we report the reaction efficiency, *k*_*eff*_≡*k*_*prod*,*Ado*_/*k*_*on*,*ATP*_, to highlight contributions specific to AMP consumption. In this regard, high *k*_*eff*_ rates generally reflect significant consumption of extant AMP pools to form Ado.

### Nucleotide transport and distribution (pools) within crowded extracellular junctions

We first discuss how nucleotide diffusion rates and distributions are influenced by physical attributes of the confined junctional geometry, including restricted diffusional volumes and electrostatic interactions between substrates, reactive enzymes and charged membrane surfaces. Independent of NDA activity, the restricted volume of the junction relative to the surrounding substrate reservoir, as well as the surface charge distribution within the junction, played key roles in shaping the nucleotide distribution. In our model, nucleotides entered the restrictive junctional domain from one of two reservoirs to emulate entrapment of species generated from an external source, such as ATP released from nearby cells. In the absence of nucleotide/surface interactions, the diffusion rate of nucleotides through the junction decreases as its radius is reduced. This is easily rationalized by noting that the substrate flux through a cylinder normal to the nucleotide concentration gradient scales proportionally to the cylinder’s cross-sectional area relative to the reservoir surface area [82]. The constriction of the substrate-accessible volume at the junction opening leads to a substantial reduction in the amount of substrate available to the enzyme within the junction compared to bulk conditions (see also [53]). For this reason, narrow junctions between cells are anticipated to limit nucleotide pools available to ATPases and ATP-gated receptors localized to extracellular junctions. We speculate therefore that estimates of ATP based on bulk (extracellular) measurements could be unrepresentative of the local ATP pools formed within the compact interstitia between cells. A strong deviation would justify the use of localized measurements of nucleotides when probing receptor activity in neural synapses for instance via microelectrodes [10].

Membrane charges can have a remarkable influence on nucleotide concentrations within junctions. As an example, we found that nucleotide concentrations for non-reactive membrane are modulated by 58% (decrease), 25% (increase) and 33% (increase) for ATP, AMP, Ado, respectively, given surface potentials of +25mV (see Fig 5). These trends are consistent with higher Ca^2+^ concentrations that have been reported along the plasma membrane of cardiac muscle cells relative to bulk cytosol, which has been attributed to the abundance of negative charges in the phospholipid membrane [83, 84].

A secondary focus of this computational study was to probe NDA-dependent modulation of steady-state nucleotide concentrations relative to those determined by junction size and electrostatic charge alone. It is understood that NDAs rapidly degrade nucleotides released in synaptic junctions [85]; hence, pulsatile release of ATP from post-synaptic neurons is followed by transient upswings in the synaptic ATP concentration that terminate within milliseconds owing to NDA degradation [10]. However, the femtoliter volume of such spaces [11] and inter-cell separations on the order of the Debye length suggest that NDA activity and resulting nucleotide pools will be sensitive to NDA colocalization, strengths of substrate/enzyme electrostatic interactions and the junction volume. Firstly, in analogy to the reduced nucleotide concentration reported at the junction/reservoir boundary, we observed substantially lower *k*_*on*,*ATP*_ rates for junction-confined CD39 relative to the bulk configuration. This behavior is easily rationalized by the smaller cross-section of the junction relative to an open system, which both reduces the concentration of substrate at the enzyme surface, as well as the accessibility of the reactive enzyme surface. Further, our predictions are consistent with classic theoretical studies relating the dynamic accessibility of gated protein active sites or substrate tunnels to observed enzyme activity [86], as demonstrated in acetylcholinesterase [87] and the PutA peripheral membrane flavoenzyme [88]. Since to a certain extent *k*_*prod*,*Ado*_ scales proportionally to *k*_*on*,*ATP*_, reduced NDA rates owing to confinement suggests that in vitro characterization of NDA activity in bulk media likely yield faster kinetics than would be expected for strongly confined systems. Our modeling results suggest that NDA confinement manifests in junctional ATP pools that were considerably smaller than the bulk, and Ado pools that were considerably larger. Based on these predictions, we anticipate that the degradation of adenosine phosphates to lower order molecules by ectonucleotidases proceeds more slowly in confined extracellular spaces relative to bulk conditions, yet under steady-state conditions larger Ado and AMP pools are evident relative to ATP. Further, this reduction in reactivity is largely determined by the reaction rate of the first species, ATP.

In contrast to the consistent rate-limiting effect of enzyme confinement on *k*_*on*,*ATP*_, the efficiency of Ado production relative to bulk varied depending on the nature of substrate/membrane interactions. We investigated this dependency assuming reflective (non-interacting) and absorbing boundary conditions on the membrane for the AMP intermediate. The latter configuration is representative of nucleotide depletion by membrane-bound enzymes or translocases. We found that efficiency was maximized when AMP did not significantly interact with the membrane (reflective). In this case, although CD39’s confinement to the junction limited its access to ATP, the membrane prevented intermediate diffusion away from CD73. This established a relatively high intermediate concentration within the junction that in turn increased *k*_*prod*,*Ado*_. In contrast, efficiency was strongly reduced when nucleotides were depleted at the surface (absorbing), as might be expected for significant nucleotide uptake by plasma membrane adenine nucleotide translocases [69]. As discussed in the next section, the reduced efficiency stemming from membranes that consume AMP could be countered by co-localizing CD39 and CD73 to promote its reaction at CD73 relative to diffusing toward the membrane. Ultimately, these findings suggest that nucleotide pools capable of activating targets such as ADP sensitive P2Y channels will be strongly regulated by the relative activity of proteins or transporters along the membrane.

Numerous biochemical processes that involve diffusing reactants rely on close spatial coupling of enzymes to promote efficient signaling. Examples of enzyme co-localization include formation of macro-molecular complexes [89, 90], confinement in molecular ‘tunnels’ [91–93], the proximal reactive sites in the sulfate-activating complex [94], in addition to metabolic substrate channeling [95–97]. We had thus expected that co-localizing NDAs within junctions would improve reaction efficiency. However, we found that close spatial coupling was advantageous only when the junction membrane significantly interacted with the intermediate. Specifically, when the membrane either absorbed the intermediate or concentrated the intermediate through attractive electrostatic interactions, smaller concentration gradients were evident at CD73 and thereby reduced *k*_*on*,*AMP*_. Co-localizing CD39 and CD73 minimized the intermediate’s access to the membrane and thus facilitated faster *k*_*on*,*AMP*_ rates than were evident at larger enzyme separations. This behavior is consistent with simulation studies by us and others [23, 46, 86] for open (bulk) systems whereby co-localization of sequential enzymes can enhance reaction rates. Based on our rationalization in the preceding paragraph, co-localization of CD39 and CD73 for a reflective membrane had minimal impact on the reaction efficiency, given that the intermediate had limited capacity to escape the reactive sites. Constructs including micelle- or viral capsid-based nanoreactors that house enzymes, or enzymes immobilized to linear or planar molecular assembles [56, 98] exhibit analogous increases in efficiency through mitigating loss of intermediates to open boundaries. These results therefore suggest that variations in NDA co-localization could provide a means to tune the relative composition of nucleotide pools within junctions, particularly for charged membranes or those with an abundance of proteins that compete for nucleotides.

A central contribution from our study is to confirm the significant role of electrostatics and intermediate channeling in facilitating coupled nucleotide hydrolysis reactions catalyzed by NDAs in nanoscale volumes. Secondarily, we demonstrate that tuning of the surface/enzyme and surface/substrate interactions can further optimize reaction rates. A third contribution of our study was to systematically characterize how electrostatic interactions influence enzyme kinetics under physiological conditions. It is clear from our simulation data that a significant membrane charge can redistribute the populations of charged substrates along the junction boundaries. Because diffusion-limited reaction rates scale proportionally to the substrate concentration gradient at the enzyme active site, it was expected that membrane charge configurations that localized substrates to the junction and its surface would enhance the reaction rate. Overall, these factors were found to significantly alter the absolute and relative concentrations of the nucleotide pools near the NDAs.

From this standpoint, we can treat our predicted *k*_*on*,*ATP*_ values as readouts for the significance of local substrate concentrations in modulating NDA activity, particularly in the context of electrostatic interactions. Such electrostatic interactions have been speculated to contribute to the formation of ‘micro-domains’ localized to the membrane surface, such as for Ca^2+^ and Na^+^ [58, 75, 99, 100] following transient fluctuations in their concentrations. These microdomains are strongly implicated in modulating the ion-dependent activation of small proteins [83]. As an example, ATP has been suggested to assume concentrations several-fold higher than the bulk cytosol, based on modeling and ATPase enzyme assays [29, 97]. To the extent that the microdomains arise exclusively from electrostatic interactions, microdomain effects would be expected to be maximal within the membrane’s electric double layer that is approximately 1 nm at physiological ionic strength.

Therefore we sought to examine extent to which electrostatic interactions contribute to microdomains under steady state conditions. We found that the reaction rate coefficient had weak dependence on the enzymes’ distance from the junction surface, regardless of ionic strength, which strongly suggests microdomains arise from a different basis. Consistent with this argument is our recent finding that ionic-strength-dependent changes in the Ca^2+^ at the membrane surface have negligible impact on SERCA Ca^2+^ affinity [101]. This confirms that localized substrate pools near the surface stem from non-equilibrium conditions, namely a net flux of substrate from the extracellular or cytosolic domains toward the membrane. For ATP, the steady-state flux toward the cytosolic side of the membrane could arise from the creatine and adenylate kinase shuttles [29, 97], while localized ATP gradients on the extracellular side could result from F1-F0 ATP synthase or translocase activity [8]. For ions such as Ca^2+^, membrane-localized gradients could arise from small inward fluxes of plasma membrane currents or leak from compartments such as the endoplasmic reticulum [83].

Our results suggest that the predominant effect of charging the membrane is to increase the concentration of ATP within the entire junction interior. This was evident in the predicted junctional ATP concentrations (see Fig A in S1 Figures), which varied significantly from that in the bulk reservoirs. This raises an interesting possibility that NDA activity could be modulated through controlling the surface charge by varying membrane lipid composition. Such variations in lipid composition and surface charge are known to occur during phagocytosis [102] and within neural synapses [70].

In addition to substrate/surface interactions, we demonstrate that electrostatic interactions between nucleotide substrates and their enzymes targets accelerate NDA activity. Favorable long-range electrostatic interactions between enzymes and substrates are well known to optimize diffusion-limited reactions in biological systems [64]. Chiefly, enzymes that bear charges complementary to their substrates typically exhibit reaction rates that are several orders of magnitude higher than rates observed with neutral species or at high ionic strengths that shield electrostatic interactions [53]. We specifically address this for CD39, CD73 and charged membranes. CD39, for example, appears to have a slightly greater density of positively-charged amino acids near the nucleotide binding domain. We would expect this positive charge center to enhance the association rate via complementary electrostatic interactions through Arg56, Lys79, Lys80, and Lys82. We qualitatively verified this hypothesis by visual inspection of the electrostatic potential of a representative CD39 structure, the NTPDase2 from *Legionella pneumophila* (PDB code 4BR7 [28]) using the Adaptive Poisson Boltzmann Solver APBS [103]. Hence we expect this to facilitate the rapid reaction, though to our knowledge rates with respect to ionic strength for this enzyme have not been reported.

Beyond the role of electrostatics in shaping *k*_*on*,*ATP*_, our results demonstrate the kinetic advantage of co-localizing charged enzymes. When the enzymes were co-localized, the influence of electrostatic interactions on the reaction rates were most strongly evident, with the fastest rates reported for closely-opposed enzymes that adopt surface charges complementary to their substrates. This finding mirrors trends observed in other coupled enzymatic processes; namely in the event that enzymes or reactive sites are sequentially aligned for coupled enzymatic reactions, electrostatic channeling of substrates is commonly exploited in nature to optimize the rate or efficiency of substrate conversion [79, 104, 105]. As an example, a computational study of the dihydrofolate reductase-thymidylate synthase (DHFR-TS) enzyme in prokaryotes has revealed that tetrahydrofolate production rates are accelerated by a patch of positively-charged amino acids between the thymidylate synthase and dihydrofolate reductase reactive sites, which facilitate transfer of the anionic dihydrofolate intermediate [25].

### Relevance to cellular physiology

ATP and its derivatives serve critical roles in cell-to-cell signaling, ion transport, intracellular signaling and cell energetics, thus it is likely that cells benefit from a well-controlled pool of these nucleotides. It is evident from our simulations that the junctional environment, as defined by its confined volume and presence of charged enzymes and membrane, dramatically alter nucleotidase activity and resultant nucleotide pools relative to an open or bulk system. Despite this, we demonstrate that nucleotidase reaction kinetics are sensitive to variations in the junctional volume and charge configurations. Moreover, the availability of nucleotide substrates is additionally subject to the activity and expression levels of nucleotide sources, including connexins and P2X7 channels, as well as competing nucleotide-dependent receptors that could include purinergic and adenosine receptors [21]. For these reasons, cells may buffer these variations by controlling CD39 and CD73 expression or co-localization. This is supported by reports that CD39 and CD73 are co-expressed in B- [20] and T-cells [19]; for the latter cells, these nucleotidases can be co-localized when CD39-expressing regulatory T-cells target CD73-expressing effector T-cells as part of their immunoregulatory function [106]. This is an addition to the rich variety of nucleotidase isoforms that differ in their substrate specificity, kinetics and products they form [13], all of which can modulate the nucleotide pool at the cell membrane.

### Limitations and future directions

In order to work with a system that was numerically solvable, we made several assumptions. Firstly, we assumed all enzymatic reactions were fast compared to the diffusion of nucleotides between reactive centers. NDAs are known to rapidly manage nucleotide pools with reaction rates on the order of 1 μM s^−1^ [30]. Since the intrinsic reaction rates of these enzymes vary considerably depending on the isoform and cell type, we assumed reaction-limited conditions for simplicity and generality.

In future studies, we might relax our assumption of a spatially- and temporally-heterogeneous membrane potentials, or consider time-dependent NDA activity and ATP sources. Additionally, our finite element modeling approach can be refined with detailed structural models of enzymes and their cellular environment, although our previous studies have indicated that when considering the native structure of the proteins, the predicted reaction rates [63] exhibit similar trends relative to those from spherical approximations. Along these lines, we have previously used finite element models to probe Ca^2+^ binding rates to myofilament proteins bound to actin chains [63, 107, 108] at atomic resolution, using mesh building software [109] applied to structures found in the Protein Databank, which could easily be extended to the NDA systems here.

### Conclusions

Sequentially-coupled enzymatic processes have been extensively probed in the literature. Our contribution in this paper complements these studies through offering insights into nucleotide signals and NDA activity within junctional interfaces between cells. Our results are consistent with the well-established notions of electrostatic channeling and co-localization accelerating reaction rates. Our approach is unique given its basis in a finite-element framework that allows for the direct incorporation of electron or confocal microscopy data, such as for serial block images of neurons or neuromuscular junctions [110], or for atomistic resolution molecular structures of NDA. In order to generalize our results, we utilized a simplified junction/spherical enzyme framework, for which we could easily vary system parameters such as distances and radii that would otherwise be difficult with structurally-detailed models.

Our simulations of steady-state NDA nucleotide hydrolysis activity in the model junctional geometry emulates the femtoliter-sized domains formed between cells. We found that confinement and high charge densities within confined domains alter nucleotide concentrations relative to the bulk solvent, independent of NDA catalytic rates. For NDAs localized to the junction, their confinement reduces enzymatic activity relative to the bulk. However, long-range nucleotide interactions with the enzymes and membrane can enhance the efficiency of product formation. Chiefly, we found that efficiency is enhanced by three factors: 1) when coupled enzymes are co-localized, 2) as confinement restricts diffusion of substrate away from the enzymes, and 3) by charge configurations that attract substrate into the junction and also towards the enzymes. We believe these findings provide new insights into how nucleotide substrate pools are managed within intercelluar interfaces and thereby control purinergic receptors and other proteins that respond to extracellular ATP. More broadly, our spatially detailed enzyme modeling could expand our capacity to probe physiological phenomena in vivo, monitor and tailor drug delivery kinetics (reviewed in [111]), and engineer biosynthetic pathways, especially those utilizing immobilized enzymes [43, 95, 112].

## Methods

### Overview

The purpose of this study was to understand the steady-state properties of nucleotide hydrolysis by NDAs in nanoscale extracellular domains. This was achieved through computer simulations of electrokinetic transport, similar to those in [53], which we modified to handle NDA reaction equilibria. The theoretical model included steady-state partial differential equations that were solved numerically using the finite element method. The equations were solved for three-dimensional geometries resembling porous materials in [53, 82]. In previous studies [113], we have found that enzyme size and overall charge are the strongest determinants of reactivity as opposed to subtle variations in shape and surface charge. Rather than attempting to simulate the intricate geometries of in vivo systems, we used representative model geometries to reduce the computational cost of the simulations, simplify interpretation of the results, and improve the reproducibility of our findings. Here, the model geometry consisted of a narrow junctional pore spanning two reservoirs, along which an ATP concentration gradient was established. We also assumed that the enzymes were spherical with uniform reactivity and charge, which we have found are reasonable approximations of similar structurally-detailed, non-uniformly charged proteins that we have examined in other studies [63, 107]. Furthermore, it is likely that all orientations of the enzymes are equally probable, in which case a sphere is a reasonable representation. We used a radius of 2 nm for the CD39 and CD73 enzymes, based on the radius of gyration for the crystal structure of the nucleotide-bound Legionella pneumophila NTPDase1 [28] with VMD [114]. Additionally, although the fully deprotonated ATP anion assumes a charge of -4 [115], we assume in accordance with physiological systems that it is coordinated with magnesium [116] and thereby assumes a net negative charge of -2.

The computational model allows for the control of key geometric, electrostatic, and kinetic parameters, so that the effects of various phenomena can be resolved. Predicted substrate gradients that developed in the materials were used to estimate reaction rate coefficients, which directly relate to the kinetics of substrate transport. Additional physics were enabled in the computational model that accounted for electrostatic interactions and surface reactions. For comparisons against bulk conditions, we assumed millimolar concentrations for the NDA enzymes.

### Model geometry

**Fig 9:**
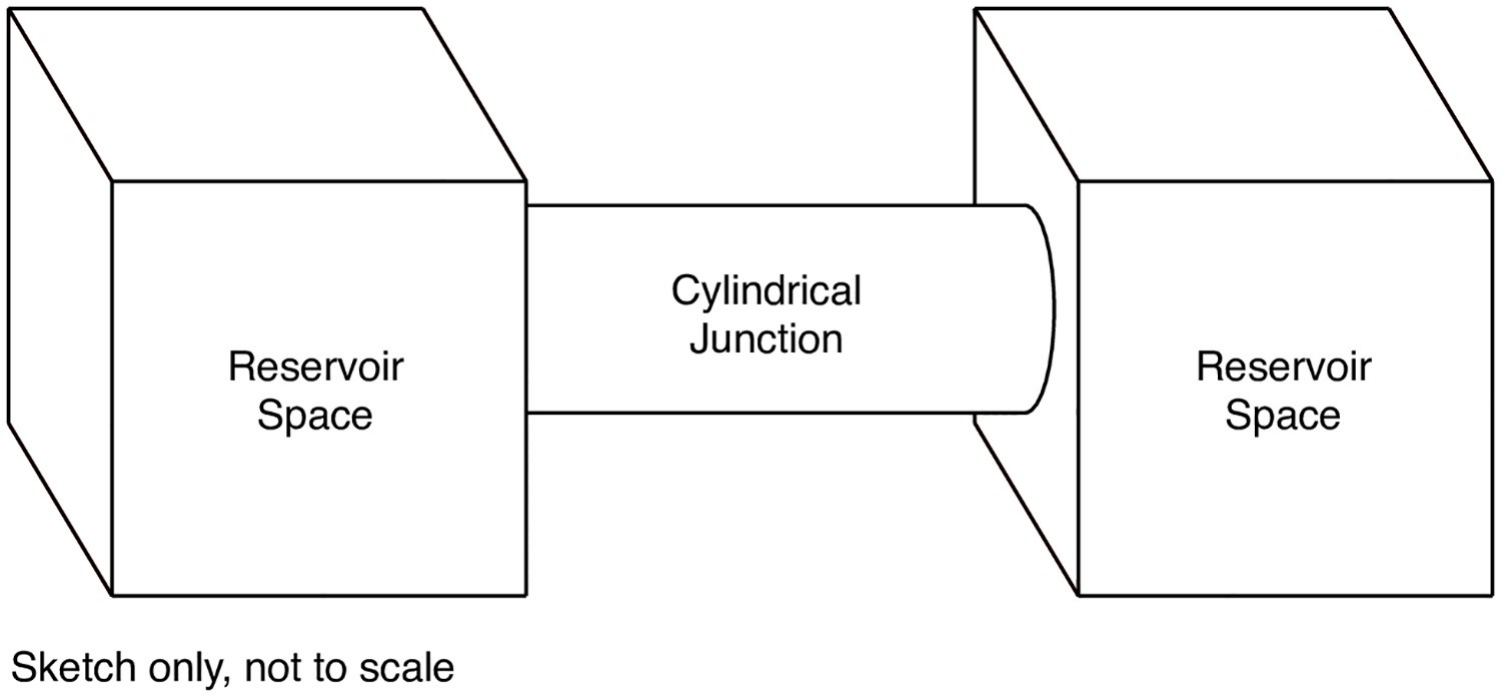
Isometric sketch of model geometry.

**Fig 10:**
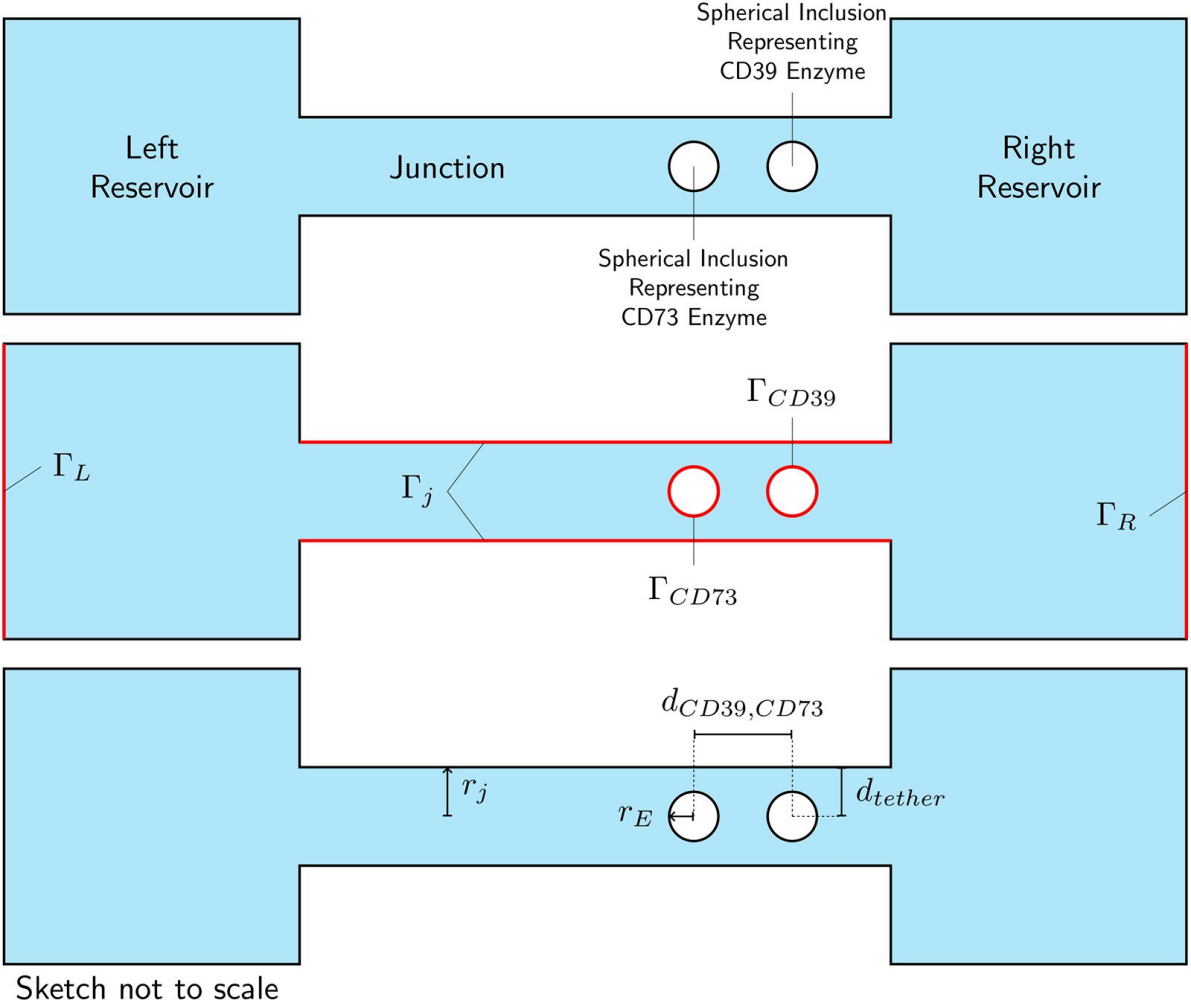
Sketch of section through model geometry. Top panel: identification of key model features. Middle panel: model boundary surfaces. Bottom panel: key dimensional parameters. For clarity, the sketches are not shown at scale.

The finite element model constructed to represent the system shown in Fig 1 consists of a cylindrical junction between two reservoirs representing the near-field non-junctional space. The cylindrical junction contains two spherical bodies representing the CD39 and CD73 enzymes. The model represents the space, through which diffusion of ATP, AMP, and Ado take place. An isometric sketch of the model is shown in Fig 9. A sketch of a cross-section through the model is shown in Fig 10, illustrating key features of the model, its boundary surfaces and key dimensional parameters.

The boundary conditions for the concentrations at model surfaces can be varied independently. The concentration of ATP is taken to be higher in the right reservoir than the left reservoir, creating a concentration gradient across the membrane. Thus, the concentration of ATP is higher at one end of the junction than the other, driving diffusion along the length of the cylinder. The concentration boundary conditions used are presented in further detail in Theory.

The electric potentials on the surface of the junction and the enzymes are designated as Ω_*junction*_, Ω_*CD*39_, and Ω_*CD*73_, respectively. These potentials can be varied independently.

The radial position of the enzymes within the junction is controlled by the parameter *d*_tether_ as shown in Fig 10. For the ‘untethered’ condition, representing a freely-diffusing isoform, the enzymes are centered within the junction (*d*_tether_ = *r*_*j*_). For the ‘tethered’ condition, representing a membrane-bound isoform, the enzymes are placed nearly in contact with the junction surface (*d*_tether_ ≈ *r*_*E*_).

### Theory

In these simulations, the enzymes were held in fixed positions while the substrates were allowed to diffuse. The problem domain was approximated as a continuum. Under these circumstances, the reaction encounter distance is just the radius of the enzyme. The enzymes are approximated as spheres.

Three species of substrate were included: ATP, AMP, and Ado. ATP is converted to the AMP product when it encounters the surface of CD39, followed by AMP’s conversion to adenosine on enzyme CD73:

$$\begin{array}{lll} \begin{array}{r} \text{ATP}\\ \text{AMP} \end{array} & \begin{array}{c} \overset{CD39}{\to }\\ \overset{CD73}{\to } \end{array} & \begin{array}{l} \text{AMP}\\ \text{Ado} \end{array} \end{array}$$ (5)

We define the concentration of a given species S as *c*_*S*_, which is an unknown spatial function to be found by solving the governing diffusion equation. The ion flux of species S is a vector field, $\vec{j_{S}}$, related to the change in concentration with respect to time through a continuity equation,

$$\begin{array}{c} \frac{\partial c_{S}}{\partial t}=-\nabla \cdot \vec{j_{S}} \end{array}$$ (6)

The diffusion of ions in a fixed electrostatic field is described by the Smoluchowski equation [117], where the flux includes both a Fickian diffusion term and a term due to the electrostatic force:

$$\begin{array}{c} {\vec{j}}_{S}=-D_{S}(\nabla c_{S}+\beta z_{S}c_{S}\nabla \Phi ) \end{array}$$ (7)

where *z*_*S*_ is the electric charge of species S, Φ is the electric potential as a scalar field, *D*_*S*_ is the Fickian diffusion coefficient for species S in the relevant media, and *β* is 1/*k*_*B*_ *T* for temperature *T* and Boltzmann constant *k*_*B*_. In this equation, the diffusion coefficient is assumed to be homogeneous and isotropic.

Using this flux in the continuity equation, the Smoluchowski equation can be written as:

$$\begin{array}{c} \frac{\partial c_{S}}{\partial t}=\nabla \cdot (D_{S}(\nabla c_{S}+\beta z_{S}c_{S}\nabla \Phi )) \end{array}$$ (8)

Under steady state conditions the concentration of species S does not vary in time, and so the governing differential equation is:

$$0=\nabla \cdot (D_{S}(\nabla c_{S}+\beta z_{S}c_{S}\nabla \Phi ))$$ (9)

To reduce the computational burden, an alternate form of the Smoluchowski equation is used. The substrate flux is expressed as:

$$\begin{array}{c} \vec{j_{S}}=-D_{S}e^{-\beta z_{S}\Phi }\nabla (e^{\beta z_{S}\Phi }c_{S}) \end{array}$$ (10)

The equivalence of these two expressions for the flux can be readily verified using the product rule for gradients. The advantage of this alternate form is that it allows for the application of the Slotboom transformation [118] [119]:

$$\begin{array}{c} \begin{array}{ccc} {\bar{D}}_{S} & = & D_{S}e^{-\beta z_{S}\Phi }\\ {\bar{c}}_{S} & = & c_{S}e^{\beta z_{S}\Phi } \end{array} \end{array}$$ (11)

After applying this transformation, the Smoluchowski equation is expressed in a form analogous to a simple Fickian diffusion equation:

$$\begin{array}{c} \nabla \cdot ({\bar{D}}_{S}\nabla ({\bar{c}}_{S}))=0 \end{array}$$ (12)

Eq 12 must be solved for each species.

We also define the integrated flux over any surface Γ as

$$\begin{array}{c} J_{S}=\int_{\Gamma }\vec{j_{S}}\cdot \hat{n}d\Gamma \end{array}$$ (13)

where $\hat{n}$ is the unit normal to the surface Γ.

The reaction kinetics at an enzyme were assumed to follow a simple rate law. For the reactions in Eq 5, the rate laws are given by

$$\begin{array}{c} \begin{array}{c} k_{CD39}c_{ATP}\equiv J_{ATP}=-J_{AMP}\\ k_{CD73}c_{AMP}\equiv J_{AMP}=-J_{Ado} \end{array} \end{array}$$ (14)

which defines reaction rate coefficients *k*_*CD*39_ = *k*_*on*,*ATP*_ = *k*_*prod*,*AMP*_ and *k*_*CD*73_ = *k*_*on*,*AMP*_ = *k*_*prod*,*Ado*_. These rate equations indicate that a given enzyme consumes one NDA species and produces another at the same rate, and this rate depends on the concentration of the consumed species. Note that the integrated flux is positive for the consumed species and negative for the produced species, as the surface normal vectors are assumed to be oriented outward.

The calculation procedure began by solving Eq 12 for $\bar{c}$ for each species, then computing the flux vector fields from the Slotboom transformation of Eq 7, then integrating the flux over the enzyme surface, and using the integrated flux to calculate the rate coefficient. The rate laws of Eq 14 were enforced at each enzyme by requiring that ${\vec{j}}_{ATP}\cdot \hat{n}=-{\vec{j}}_{AMP}\cdot \hat{n}$ on the surface of CD39, and ${\vec{j}}_{AMP}\cdot \hat{n}=-{\vec{j}}_{Ado}\cdot \hat{n}$ on the surface of CD73.

A summary of concentration boundary conditions applied to the model is presented in Table 2.

**Table 2:** Boundary conditions for the nanoporous system. Boundary surfaces are as illustrated in Fig 10. The boundary condition for AMP in the junctional surface may assume reflective (nonreactive) or absorbing (reactive) conditions. Here, $j_{S}={\vec{j}}_{S}\cdot \hat{n}$.

Boundary Surface	ATP	AMP	Ado
Γ_*CD*39_	*c*_*ATP*_ = 0	*j*_*AMP*_ = −*j*_*ATP*_	*j*_*Ado*_ = 0
Γ_*CD*73_	*j*_*ATP*_ = 0	*c*_*AMP*_ = 0	*j*_*Ado*_ = −*j*_*AMP*_
Γ_*R*_	*c*_*ATP*_ = *C*_0_	*c*_*AMP*_ = 0	*c*_*Ado*_ = 0
Γ_*L*_	*c*_*ATP*_ = 0	*c*_*AMP*_ = 0	*c*_*Ado*_ = 0
Γ_*j*_	*j*_*ATP*_ = 0	*j*_*AMP*_ = 0 or *c*_*AMP*_ = 0	*j*_*Ado*_ = 0

The solution of Eq 12 requires knowledge of the electric potential Φ throughout the model. The electric potential is found by solving the linearized Poisson-Boltzmann equation:

$$\begin{array}{c} \nabla^{2}\Phi =\kappa^{2}\Phi \end{array}$$ (15)

where *κ* is the Debye-Hückel parameter which is proportional to square root of ionic strength (I). For pure aqueous solvent, *κ* = 0 and therefore Eq 15 reduces to the Poisson equation commonly used in electrostatics. The electrostatic boundary conditions for Eq 15 consist of setting the electric potential to zero at both the left and right reservoir boundaries, and the electric potential along the junction and enzymes are set in accordance with Table 3 and Eq 16, the Graham equation:

$$\begin{array}{c} \sigma =\sqrt{8c_{0}\epsilon \epsilon_{0}k_{B}T}\text{sinh}(\frac{e\phi_{0}}{2k_{B}T}) \end{array}$$ (16)

**Table 3:** Summary of cases run.

Fig (Sect)	qATP/CD39	qAMP/CD73	junction	λ_*D*_
Fig 2	-2/0	-1/0	0	NA
Fig 3	-2/0	-1/0	0	NA
Fig 4	-2/0	-1/0	0	NA
Fig 6	-2/var.	-1/0	var	10 nm
Fig 7	-2/25.0 mV	-1/var	var	10 nm
Fig 8a	-2/0	-1/0	var.	var.
Fig 8b	-2/25.0 mV	-1/0	var.	var.
Fig 5	-2/0	-1/var.	var.	10 nm

### Numerical approach

Methodologies are generally as described in Sun et al., [53].

The system of partial differential equations and boundary conditions described above were solved numerically using the Finite Element Method. The open-source finite element package FEniCS [120], version 2017.2.0 was used to conduct the simulations. This software is publicly available at https://fenicsproject.org.

A second-order polynomial (Lagrange) basis set was used for all finite elements. The differential equations to be solved were all linear, so no nonlinear solution schemes were required. Various linear solvers and preconditioners were employed in order to obtain solutions.

Python-based analysis routines were used to set up, solve, and post-process the finite element models. All code written in support of this publication is publicly available at https://bitbucket.org/pkhlab/pkh-lab-analyses. Simulation input files and generated data are available upon request. Although all simulation data generated in this paper can be reproduced from the python routines provided in the bitbucket repository, we have also included the raw data generated in this project as a tarball at https://doi.org/10.5281/zenodo.3711649.

## Supporting information

S1 Figures Additional Results Figures. (PDF)

S1 Text Analogy between Fickian diffusion and the Laplace equation in electrostatics, additional validation and comparisons. With supporting figures.
(PDF)

S2 Text Mathematical notation and abbreviations. (PDF)

## References

[1] Vendelin M, Birkedal R. Anisotropic diffusion of fluorescently labeled ATP in rat cardiomyocytes determined by raster image correlation spectroscopy. AJP Cell Physiol. 2008;295(5):C1302–C1315. 10.1152/ajpcell.00313.2008 PMC2584976 18815224

[2] Somlyo AP, Somlyo AV. Signal transduction and regulation in smooth muscle. Nature. 1994;372(6503):231–236. 10.1038/372231a0 7969467

[3] Shen-Orr SS, Milo R, Mangan S, Alon U. Network motifs in the transcriptional regulation network of Escherichia coli. Nature Genetics. 2002;31(1):64 10.1038/ng881 11967538

[4] Davidson EH, Rast JP, Oliveri P, Ransick A, Calestani C, Yuh CH, et al A genomic regulatory network for development. Science (New York, NY). 2002;295(5560):1669–1678. 10.1126/science.1069883 11872831

[5] Srere PA. COMPLEXES OF SEQUENTIAL METABOLIC ENZYMES. Annual Review of Biochemistry. 1987;56(1):89–124. 10.1146/annurev.bi.56.070187.000513 2441660

[6] North RA. Molecular Physiology of P2X Receptors. Physiol Rev. 2002;82(4):1013–1067. 10.1152/physrev.00015.2002 12270951

[7] von Kugelgen I, Hoffmann K. Pharmacology and structure of P2Y receptors. Neuropharmacology. 2016;104:50–61. 10.1016/j.neuropharm.2015.10.030 26519900

[8] Ashley TA, Misra UK, Roy JA, Goodman MD, Pizzo SV, Kenan DJ, et al Endothelial cell surface F1-FO ATP synthase is active in ATP synthesis and is inhibited by angiostatin. Proc Natl Acad Sci. 2002;98(12):6656–6661. 10.1073/pnas.131067798 PMC34409 11381144

[9] Khakh BS. Molecular physiology of P2X receptors and ATP signalling at synapses. Nat Rev Neurosci. 2001;. 10.1038/35058521 11256077

[10] Lalo U, Palygin O, Rasooli-Nejad S, Andrew J, Haydon PG, Pankratov Y. Exocytosis of ATP From Astrocytes Modulates Phasic and Tonic Inhibition in the Neocortex. PLoS Biol. 2014;12(1):e1001747 10.1371/journal.pbio.1001747 24409095 PMC3883644

[11] Bito H. The chemical biology of synapses and neuronal circuits. Nat Chem Biol. 2010;6(8):560–563. 10.1038/nchembio.408 20644538

[12] Deaglio S, Robson SC. Ectonucleotidases as Regulators of Purinergic Signaling in Thrombosis, Inflammation, and Immunity. Advances in Pharmacology. 2011;61:301–332. 10.1016/B978-0-12-385526-8.00010-2 21586363 PMC5879773

[13] ZIMMERMANN H. BIOCHEMISTRY, LOCALIZATION AND FUNCTIONAL ROLES OF ECTO-NUCLEOTIDASES IN THE NERVOUS SYSTEM. Prog Neurobiol. 1996;49(6):589–618. 10.1016/0301-0082(96)00026-3 8912394

[14] Giuliani AL, Sarti AC, Di Virgilio F. Extracellular nucleotides and nucleosides as signalling molecules. Immunol Lett. 2019;205:16–24. 10.1016/j.imlet.2018.11.006 30439478

[15] Aliev MK, Tikhonov AN. Random walk analysis of restricted metabolite diffusion in skeletal myofibril systems. Mol Cell Biochem. 2004;256-257(1-2):257–66. 10.1023/B:MCBI.0000009873.37245.54 14977186

[16] Aliev M, Guzun R, Karu-Varikmaa M, Kaambre T, Wallimann T, Saks V. Molecular System Bioenergics of the Heart. Int J Mol Sci. 2011;12(12):9296–9331. 10.3390/ijms12129296 22272134 PMC3257131

[17] Kukulski F, Levesque SA, Sevigny J. Impact of Ectoenzymes on P2 and P1 Receptor Signaling. Advances in Pharmacology. 2011;61:263–299. 10.1016/B978-0-12-385526-8.00009-6 21586362

[18] Goueli SA, Hsiao K. Monitoring and characterizing soluble and membrane-bound ectonucleotidases CD73 and CD39. PLOS ONE. 2019;14(10):e0220094 10.1371/journal.pone.0220094 31652269 PMC6814236

[19] Deaglio S, Dwyer KM, Gao W, Friedman D, Usheva A, Erat A, et al Adenosine generation catalyzed by CD39 and CD73 expressed on regulatory T cells mediates immune suppression. Journal of Experimental Medicine. 2007;204(6):1257–1265. 10.1084/jem.20062512 17502665 PMC2118603

[20] Saze Z, Schuler PJ, Hong CS, Cheng D, Jackson EK, Whiteside TL. Adenosine production by human B cells and B cell–mediated suppression of activated T cells. Blood. 2013;122(1):9–18. 10.1182/blood-2013-02-482406 23678003 PMC3701906

[21] Abbracchio MP, Burnstock G, Verkhratsky A, Zimmermann H. Purinergic signalling in the nervous system: an overview. Trends in Neurosciences. 2009;32(1):19–29. 10.1016/j.tins.2008.10.001 19008000

[22] Tyson JJ, Novak B. Functional Motifs in Biochemical Reaction Networks. Annual Review of Physical Chemistry. 2010;61(1):219–240. 10.1146/annurev.physchem.012809.103457 20055671 PMC3773234

[23] Eun C, Kekenes-Huskey PM, Metzger VT, McCammon JA. A model study of sequential enzyme reactions and electrostatic channeling. J Chem Phys. 2014;140(10):105101 10.1063/1.4867286 24628210 PMC3977847

[24] Yamaguchi N, Prosser BL, Ghassemi F, Xu L, Pasek DA, Eu JP, et al Modulation of sarcoplasmic reticulum Ca 2+ release in skeletal muscle expressing ryanodine receptor impaired in regulation by calmodulin and S100A1. Am J Physiol Physiol. 2011;300(5):C998–C1012. 10.1152/ajpcell.00370.2010 PMC3093939 21289290

[25] Metzger VT, Eun C, Kekenes-Huskey PM, Huber G, McCammon JA. Electrostatic channeling in P. falciparum DHFR-TS: Brownian dynamics and smoluchowski modeling. Biophys J. 2014;107(10):2394–2402. 10.1016/j.bpj.2014.09.039 25418308 PMC4241442

[26] Dorsaz N, De Michele C, Piazza F, De Los Rios P. Diffusion-limited reactions in crowded environments. Phys Rev. 2010; p. 2012. 10.1103/PhysRevLett.105.120601 20867619

[27] Kekenes-Huskey PM, Scott CE, Atalay S. Quantifying the Influence of the Crowded Cytoplasm on Small Molecule Diffusion. J Phys Chem B. 2016;120(33):8696–8706. 10.1021/acs.jpcb.6b03887 27327486 PMC8867400

[28] Zebisch M, Krauss M, Schafer P, Lauble P, Strater N. Crystallographic snapshots along the reaction pathway of nucleoside triphosphate diphosphohydrolases. Structure. 2013;21(8):1460–75. 10.1016/j.str.2013.05.016 23830739

[29] Alekseev AE, Reyes S, Selivanov VA, Dzeja PP, Terzic A. Compartmentation of membrane processes and nucleotide dynamics in diffusion-restricted cardiac cell microenvironment. J Mol Cell Cardiol. 2012;52(2):401–409. 10.1016/j.yjmcc.2011.06.007 21704043 PMC3264845

[30] Sandefur CI, Boucher RC, Elston TC. Mathematical model reveals role of nucleotide signaling in airway surface liquid homeostasis and its dysregulation in cystic fibrosis. Proc Natl Acad Sci. 2017;114(35):E7272–E7281. 10.1073/pnas.1617383114 28808008 PMC5584404

[31] Elcock AH. Models of macromolecular crowding effects and the need for quantitative comparisons with experiment. Curr Opin Struct Biol. 2010;20(2):196–206. 10.1016/j.sbi.2010.01.008 20167475 PMC2854290

[32] Chen WW, Niepel M, Sorger PK. Classic and contemporary approaches to modeling biochemical reactions. Genes, Development. 2010;24(17):1861–1875. 10.1101/gad.1945410 20810646 PMC2932968

[33] Arkin A, Ross J. Computational functions in biochemical reaction networks. Biophysical Journal. 1994;67(2):560–578. 10.1016/S0006-3495(94)80516-8 7948674 PMC1225399

[34] Jeong H, Tombor B, Albert R, Oltvai ZN, Barabasi AL. The large-scale organization of metabolic networks. Nature. 2000;407(6804):651 10.1038/35036627 11034217

[35] Shinar G, Feinberg M. Structural Sources of Robustness in Biochemical Reaction Networks. Science. 2010;327(5971):1389–1391. 10.1126/science.1183372 20223989

[36] Papin JA, Reed JL, Palsson BO. Hierarchical thinking in network biology: the unbiased modularization of biochemical networks. Trends in Biochemical Sciences. 2004;29(12):641–647. 10.1016/j.tibs.2004.10.001 15544950

[37] Schreiber G, Haran G, Zhou HX. Fundamental Aspects of Protein-Protein Association Kinetics. Chem Rev. 2009;109(3):839–860. 10.1021/cr800373w 19196002 PMC2880639

[38] Shutova VV, Yusipovich AI, Parshina EY, Zakharkin DO, Revin VV. Effect of particle size on the enzymatic hydrolysis of polysaccharides from ultrafine lignocellulose particles. Applied Biochemistry and Microbiology. 2012;48(3):312–317. 10.1134/S000368381203012X 22834308

[39] Bailey JE, Cho YK. Immobilization of glucoamylase and glucose oxidase in activated carbon: Effects of particle size and immobilization conditions on enzyme activity and effectiveness. Biotechnology and Bioengineering. 1983;25(8):1923–1935. 10.1002/bit.260250803 18551539

[40] Jia H, Zhu G, Wang P. Catalytic behaviors of enzymes attached to nanoparticles: the effect of particle mobility. Biotechnology and Bioengineering. 2003;84(4):406–414. 10.1002/bit.10781 14574697

[41] Zhou HX. How do biomolecular systems speed up and regulate rates? Phys Biol. 2005;2(3):R1–R25. 10.1088/1478-3975/2/3/R01 16224118

[42] Alberty RA, Hammes GG. Application of the Theory of Diffusion-controlled Reactions to Enzyme Kinetics. The Journal of Physical Chemistry. 1958;62(2):154–159. 10.1021/j150560a005

[43] Schoffelen S, van Hest JCM. Multi-enzyme systems: bringing enzymes together in vitro. Soft Matter. 2012;. 10.1039/C1SM06452E

[44] Garcia GJM, Picher M, Zuo P, Okada SF, Lazarowski ER, Button B, et al Computational model for the regulation of extracellular ATP and adenosine in airway epithelia. Sub-cellular biochemistry. 2011;55:51–74. 10.1007/978-94-007-1217-1_3 21560044

[45] Roa R, Siegl T, Kim WK, Dzubiella J. Product interactions and feedback in diffusion-controlled reactions. J Chem Phys. 2018;148(6):1–2. 10.1063/1.5016608 29448770

[46] Kuzmak A, Carmali S, von Lieres E, Russell AJ, Kondrat S. Can enzyme proximity accelerate cascade reactions? Sci Rep. 2019;9(1):1–2. 10.1038/s41598-018-37034-3 30679600 PMC6345930

[47] Eun C, Kekenes-Huskey PM, McCammon JA. Influence of neighboring reactive particles on diffusion-limited reactions. J Chem Phys. 2013;139(4):044117 10.1063/1.4816522 23901970 PMC3745503

[48] Zhou HX. Rate theories for biologists. Quarterly reviews of biophysics. 2010;43(2):219–93. 10.1017/S0033583510000120 20691138 PMC3540998

[49] Kekenes-Huskey PM, Eun C, McCammon JA. Enzyme localization, crowding, and buffers collectively modulate diffusion-influenced signal transduction: Insights from continuum diffusion modeling. J Chem Phys. 2015;143(9):094103 10.1063/1.4929528 26342355 PMC4560719

[50] Galanti M, Fanelli D, Traytak SD, Piazza F. Theory of diffusion-influenced reactions in complex geometries. Phys Chem Chem Phys. 2016;18:15950–15954. 10.1039/C6CP90149B 27241805

[51] Kekenes-Huskey PM, Gillette A, Hake J, McCammon JA. Finite-element estimation of protein ligand association rates with post-encounter effects: applications to calcium binding in troponin C and SERCA. Computational Science Discovery. 2012;5(1):014015 10.1088/1749-4699/5/1/014015 23293662 PMC3535444

[52] Huang YmM, Huber GA, Wang N, Minteer SD, McCammon JA. Brownian dynamic study of an enzyme metabolon in the TCA cycle: Substrate kinetics and channeling. Protein Sci. 2018;27(2):463–471. 10.1002/pro.3338 29094409 PMC5775167

[53] Sun B, Blood R, Atalay S, Colli D, Rankin SE, Knutson BL, et al Simulation-based characterization of electrolyte and small molecule diffusion in oriented mesoporous silica thin films. chemrxivorg. 2017; p. 1–2.

[54] Rice. Diffusion-Controlled Reactions in Solution. Compr Chem Kinet. 1985;25(C):3–46.

[55] Berg HC, Purcell EM. Physics of chemoreception. Biophysical Journal. 1977;20(2):193–219. 10.1016/S0006-3495(77)85544-6 911982 PMC1473391

[56] Lu A, O’Reilly RK. Advances in nanoreactor technology using polymeric nanostructures. Curr Opin Biotechnol. 2013;24(4):639–645. 10.1016/j.copbio.2012.11.013 23270737

[57] Blatter LA, Niggli E. Confocal nearmmembrane detection of calcium in cardiac myocytes. Cell Calcium. 1998; p. 1–2. 10.1016/s0143-4160(98)90023-9 9681190

[58] Verdonck F, Mubagwa K, Sipido KR. [Na+] in the subsarcolemmal fuzzy space and modulation of [Ca2+]i and contraction in cardiac myocytes. Cell Calcium. 2004;35(6):603–612. 10.1016/j.ceca.2004.01.014 15110150

[59] Salgin S, Salgin U, Bahadir S. Zeta Potentials and Isoelectric Points of Biomolecules: The Effects of Ion Types and Ionic Strengths. Int J Electrochem Sci. 2012;7:12404–12414.

[60] et al C. The *β*/*α* peak height ratio of ATP. A measure of free [Mg2+] using31P NMR. Journal of Biological Chemistry. 1996;271(35):21142–21150. 10.1074/jbc.271.35.21142 8702884

[61] Klausen LH, Fuhs T, Dong M. Mapping surface charge density of lipid bilayers by quantitative surface conductivity microscopy. Nat Commun. 2016;7(1):12447 10.1038/ncomms12447 27561322 PMC5007656

[62] Moyne C, Murad MA. A Two-Scale Model for Coupled Electro-Chemo-Mechanical Phenomena and Onsager’s Reciprocity Relations in Expansive Clays: II Computational Validation. Transp porous media. 2006;62(1):13–56. 10.1007/s11242-005-1291-7

[63] Kekenes-Huskey PM, Gillette AK, McCammon JA. Predicting the influence of long-range molecular interactions on macroscopic-scale diffusion by homogenization of the Smoluchowski equation. J Chem Phys. 2014;140(17):174106 10.1063/1.4873382 24811624 PMC4032425

[64] Schreiber G, Fersht AR. Rapid, electrostatically assisted association of proteins. Nat Struct Mol Biol. 1996;3(5):427–431. 10.1038/nsb0596-427 8612072

[65] Vijayakumar M, Wong KY, Schreiber G, Fersht AR, Szabo A, Zhou HX. Electrostatic enhancement of diffusion-controlled protein-protein association: comparison of theory and experiment on barnase and barstar. Journal of Molecular Biology. 1998;278(5):1015–1024. 10.1006/jmbi.1998.1747 9600858

[66] Selzer T, Schreiber G. Predicting the rate enhancement of protein complex formation from the electrostatic energy of interaction. Journal of Molecular Biology. 1999;287(2):409–419. 10.1006/jmbi.1999.2615 10080902

[67] Khakh BS, North RA. Neuromodulation by extracellular ATP and P2X receptors in the CNS. Neuron. 2012; p. 1–2. 10.1016/j.neuron.2012.09.024 PMC4064466 23040806

[68] Moser TL, Kenan DJ, Ashley TA, Roy JA, Goodman MD, Misra UK, et al Endothelial cell surface F1-FO ATP synthase is active in ATP synthesis and is inhibited by angiostatin. Proc Natl Acad Sci. 2001;98(12):6656–6661. 10.1073/pnas.131067798 11381144 PMC34409

[69] Cardouat G, Duparc T, Fried S, Perret B, Najib S, Martinez LO. Ectopic adenine nucleotide translocase activity controls extracellular ADP levels and regulates the F 1 -ATPase-mediated HDL endocytosis pathway on hepatocytes. Biochim Biophys Acta—Mol Cell Biol Lipids. 2017;1862(9):832–841. 10.1016/j.bbalip.2017.05.005 28504211

[70] Choquet D, Triller A. The dynamic synapse. Neuron. 2013;80(3):691–703. 10.1016/j.neuron.2013.10.013 24183020

[71] Schafer DP, Lehrman EK, Stevens B. The quad-partite synapse: Microglia-synapse interactions in the developing and mature CNS. Glia. 2013;61(1):24–36. 10.1002/glia.22389 22829357 PMC4082974

[72] Kittel A, Csapó ZS, Csizmadia E, Jackson SW, Robson SC. Co-localization of P2Y1 receptor and NTPDase1/CD39 within caveolae in human placenta. Eur J Histochem. 2004;48(3):253–9. 15590415

[73] Joseph SM, Buchakjian MR, Dubyak GR. Colocalization of ATP Release Sites and Ecto-ATPase Activity at the Extracellular Surface of Human Astrocytes. J Biol Chem. 2003;278(26):23331–23342. 10.1074/jbc.M302680200 12684505

[74] Garcia-Marcos M, Dehaye JP, Marino A. Membrane compartments and purinergic signalling: the role of plasma membrane microdomains in the modulation of P2XR-mediated signalling. FEBS J. 2009;276(2):330–340. 10.1111/j.1742-4658.2008.06794.x 19076211

[75] Winslow RL, Greenstein JL. Cardiac myocytes and local signaling in nano-domains. Prog Biophys Mol Biol. 2011;107(1):48–59. 10.1016/j.pbiomolbio.2011.06.005 21718716 PMC3190073

[76] Putzel GG, Tagliazucchi M, Szleifer I. Nonmonotonic Diffusion of Particles Among Larger Attractive Crowding Spheres. Phys Rev Lett. 2014;113(13):138302 10.1103/PhysRevLett.113.138302 25302920 PMC4670031

[77] Dix JA, Verkman AS. Crowding Effects on Diffusion in Solutions and Cells. Annu Rev Biophys. 2008;37(1):247–263. 10.1146/annurev.biophys.37.032807.125824 18573081

[78] Balbo J, Mereghetti P, Herten DP, Wade RC. The Shape of Protein Crowders is a Major Determinant of Protein Diffusion. Biophys J. 2013;104(7):1576–1584. 10.1016/j.bpj.2013.02.041 23561534 PMC3617426

[79] Elcock AH, McCammon JA. Evidence for electrostatic channeling in a fusion protein of malate dehydrogenase and citrate synthase. Biochemistry. 1996;35(39):12652–12658. 10.1021/bi9614747 8841108

[80] Madry C, Arancibia-Carcamo IL, Kyrargyri V, Chan VTT, Hamilton NB, Attwell D. Effects of the ecto-ATPase apyrase on microglial ramification and surveillance reflect cell depolarization, not ATP depletion. Proc Natl Acad Sci. 2018; p. 201715354. 10.1073/pnas.1715354115 29382767 PMC5816168

[81] Sanz JM, Virgilio FD. Kinetics and Mechanism of ATP-Dependent IL-1 Release from Microglial Cells. J Immunol. 2000;164(9):4893–4898. 10.4049/jimmunol.164.9.4893 10779799

[82] Wagh P, Zhang X, Blood R, Kekenes-Huskey P, Rajapaksha P, Wei Y, et al Increasing Salt Rejection of Polybenzimidazole Nanofiltration Membranes via the Addition of Immobilized and Aligned Aquaporins. Processes. 2019;7(2):76 10.3390/pr7020076 31179235 PMC6550480

[83] Bers DM. Cardiac excitation-contraction coupling. Nature. 2002;415(6868):198–205. 10.1038/415198a 11805843

[84] Tanskanen AJ, Greenstein JL, Chen A, Sun SX, Winslow RL. Protein geometry and placement in the cardiac dyad influence macroscopic properties of calcium-induced calcium release. Biophys J. 2007;92(10):3379–3396. 10.1529/biophysj.106.089425 17325016 PMC1853149

[85] Duarte-Araujo M, Nascimento C, Timoteo MA, Magalhaes-Cardoso MT, Correia-De-Sa P. Relative contribution of ecto-ATPase and ecto-ATPDase pathways to the biphasic effect of ATP on acetylcholine release from myenteric motoneurons. Br J Pharmacol. 2009;156(3):519–533. 10.1111/j.1476-5381.2008.00058.x 19154428 PMC2697673

[86] Barreda JL, Zhou HX. Theory and simulation of diffusion-influenced, stochastically gated ligand binding to buried sites. J Chem Phys. 2011;. 10.1063/1.3645000 22010732 PMC3215080

[87] Zhou HXHX, Wlodek STST, McCammon JAJA. Conformation gating as a mechanism for enzyme specificity. Proc Natl Acad Sci. 1998;95(16):9280–9283. 10.1073/pnas.95.16.9280 9689071 PMC21329

[88] Singh H, Arentson BW, Becker DF, Tanner JJ. Structures of the PutA peripheral membrane flavoenzyme reveal a dynamic substrate-channeling tunnel and the quinone-binding site. Proc Natl Acad Sci. 2014;111(9):3389–3394. 10.1073/pnas.1321621111 24550478 PMC3948300

[89] Gao Y, Roberts CC, Toop A, Chang CeA, Wheeldon I. Mechanisms of Enhanced Catalysis in Enzyme-DNA Nanostructures Revealed through Molecular Simulations and Experimental Analysis. ChemBioChem. 2016;17(15):1430–1436. 10.1002/cbic.201600392 27173175

[90] Roberts CC, Chang CeA. Modeling of Enhanced Catalysis in Multienzyme Nanostructures: Effect of Molecular Scaffolds, Spatial Organization, and Concentration. J Chem Theory Comput. 2015;11(1):286–292. 10.1021/ct5007482 26574226

[91] Hilario E, Caulkins BG, Huang YMM, You W, Chang CEA, Mueller LJ, et al Visualizing the tunnel in tryptophan synthase with crystallography: Insights into a selective filter for accommodating indole and rejecting water. Biochim Biophys Acta—Proteins Proteomics. 2016;1864(3):268–279. 10.1016/j.bbapap.2015.12.006 PMC4732270 26708480

[92] Singh H, Arentson BW, Becker DF, Tanner JJ. Structures of the PutA peripheral membrane flavoenzyme reveal a dynamic substrate-channeling tunnel and the quinone-binding site. Proc Natl Acad Sci. 2014;111(9):3389–3394. 10.1073/pnas.1321621111 24550478 PMC3948300

[93] Luo M, Christgen S, Sanyal N, Arentson BW, Becker DF, Tanner JJ. Evidence That the C-Terminal Domain of a Type B PutA Protein Contributes to Aldehyde Dehydrogenase Activity and Substrate Channeling. Biochemistry. 2014;53(35):5661–5673. 10.1021/bi500693a 25137435 PMC4159212

[94] Cheng Y, Chang CeA, Yu Z, Zhang Y, Sun M, Leyh TS, et al Diffusional Channeling in the Sulfate-Activating Complex: Combined Continuum Modeling and Coarse-Grained Brownian Dynamics Studies. Biophys J. 2008;95(10):4659–4667. 10.1529/biophysj.108.140038 18689458 PMC2576392

[95] Chen AH, Silver PA. Designing biological compartmentalization. Trends Cell Biol. 2012;. 10.1016/j.tcb.2012.07.002 22841504

[96] Conrado RJ, Varner JD, DeLisa MP. Engineering the spatial organization of metabolic enzymes: mimicking nature’s synergy. Curr Opin Biotechnol. 2008; p. 1–2. 18725290 10.1016/j.copbio.2008.07.006

[97] Selivanov VA, Krause S, Roca J, Cascante M. Modeling of Spatial Metabolite Distributions in the Cardiac Sarcomere. Biophys J. 2007;92(10):3492–3500. 10.1529/biophysj.106.101352 17325002 PMC1853159

[98] Anwar MZ, Kim DJ, Kumar A, Patel SKS, Otari S, Mardina P, et al SnO 2 hollow nanotubes: a novel and efficient support matrix for enzyme immobilization. Sci Rep. 2017;(10):1–11. 29127386 10.1038/s41598-017-15550-y PMC5681633

[99] McCarron JG, Chalmers S, Olson ML, Girkin JM. Subplasma membrane Ca2+ signals. IUBMB Life. 2012;64(7):573–585. 10.1002/iub.1032 22653514 PMC3638344

[100] Aronsen JM, Swift F, Sejersted OM. Cardiac sodium transport and excitation contraction coupling. J Mol Cell Cardiol. 2013;61(C):11–19. 10.1016/j.yjmcc.2013.06.003 23774049

[101] Sun B, Stewart BD, Kucharski AN, Kekenes-Huskey PM. Thermodynamics of Cation Binding to the Sarcoendoplasmic Reticulum Calcium ATPase Pump and Impacts on Enzyme Function. J Chem Theory Comput. 2019; p. acs.jctc.8b01312. 10.1021/acs.jctc.8b01312 PMC7336277 30807147

[102] Yeung T, Grinstein S. Lipid signaling and the modulation of surface charge during phagocytosis. Immunol Rev. 2007;219(1):17–36. 10.1111/j.1600-065X.2007.00546.x 17850479

[103] Holst M, Baker N, Wang F. Adaptive multilevel finite element solution of the Poisson-Boltzmann equation I. Algorithms and examples. J Comput Chem. 2000;21(15):1319–1342. 10.1002/1096-987X(20001130)21:15<1319::AID-JCC1>3.0.CO;2-8

[104] Elcock AH, Huber GA, McCammon JA. Electrostatic Channeling of Substrates between Enzyme Active Sites. Biochemistry. 1997;36(51):16049–16058. 10.1021/bi971709u 9405038

[105] Elcock AH, Potter MJ, Matthews DA, Knighton DR, McCammon JA. Electrostatic Channeling in the Bifunctional Enzyme Dihydrofolate Reductase-thymidylate Synthase. J Mol Biol. 1996;262(3):370–374. 10.1006/jmbi.1996.0520 8845002

[106] Gourdin ea. Autocrine Adenosine regulates tumor polyfunctional CD73+CD4+ effector T cells devoid of immune checkpoints. Cancer Research. 2018;78(13):2405–2017. 10.1158/0008-5472.CAN-17-2405 29559470

[107] Kekenes-Huskey PM, Gillette A, Hake J, McCammon JA. Finite Element Estimation of Protein-Ligand Association Rates with Post-Encounter Effects. Comput Sci Discov. 2012;5(1):0–20. 10.1088/1749-4699/5/1/014015 PMC3535444 23293662

[108] Kekenes-Huskey PM, Liao T, Gillette AK, Hake JE, Zhang Y, Michailova AP, et al Molecular and subcellular-scale modeling of nucleotide diffusion in the cardiac myofilament lattice. Biophys J. 2013;105(9):2130–2140. 10.1016/j.bpj.2013.09.020 24209858 PMC3835335

[109] Liao T, Zhang Y, Kekenes-Huskey PM, Cheng Y, Michailova A, McCulloch AD, et al Multi-core CPU or GPU-accelerated Multiscale Modeling for Biomolecular Complexes. Mol Based Math Biol. 2013;1:164–179. 10.2478/mlbmb-2013-0009 PMC3858848 24352481

[110] Cheng Y, Suen JK, Radić Z, Bond SD, Holst MJ, McCammon JA. Continuum simulations of acetylcholine diffusion with reaction-determined boundaries in neuromuscular junction models. Biophys Chem. 2007 10.1016/j.bpc.2007.01.003 17307283 PMC2040065

[111] Savage DJ, Liu X, Curley SA, Ferrari M, Serda RE. Porous silicon advances in drug delivery and immunotherapy. Curr Opin Pharmacol. 2013;13(5):834–841. 10.1016/j.coph.2013.06.006 23845260 PMC3795885

[112] Lizana L, Konkoli Z, Orwar O. Tunable Filtering of Chemical Signals in a Simple Nanoscale Reaction-Diffusion Network. J Phys Chem B. 2007;111(22):6214–6219. 10.1021/jp068313p 17497911

[113] Kekenes-Huskey PM, Gillette AK, McCammon JA. Predicting the influence of long-range molecular interactions on macroscopic-scale diffusion by homogenization of the Smoluchowski equation. The Journal of Chemical Physics. 2014;140(17):174106 10.1063/1.4873382 24811624 PMC4032425

[114] Humphrey W, Dalke A, Schulten K. VMD—Visual Molecular Dynamics. Journal of Molecular Graphics. 1996;14:33–38. 10.1016/0263-7855(96)00018-5 8744570

[115] Alberty RA, Goldberg RN. Standard thermodynamic formation properties for the adenosine 5’-triphosphate series. Biochemistry. 1992;31(43):10610–10615. 10.1021/bi00158a025 1420176

[116] Romani AMP. Cellular magnesium homeostasis. Arch Biochem Biophys. 2011;512(1):1–23. 10.1016/j.abb.2011.05.010 21640700 PMC3133480

[117] Song Y, Zhang Y, Shen T, Bajaj CL, McCammon JA, Baker NA. Finite Element Solution of the Steady-State Smoluchowski Equation for Rate Constant Calculations. Biophysical Journal. 2004;86(4):2017–2029. 10.1016/S0006-3495(04)74263-0 15041644 PMC1304055

[118] Slotboom JW. Computer-aided two-dimensional analysis of bipolar transistors. IEEE Transactions on Electron Devices. 1973;20(8):669–679. 10.1109/T-ED.1973.17727

[119] Lu B, Holst MJ, Andrew McCammon J, Zhou YC. Poisson–Nernst–Planck equations for simulating biomolecular diffusion–reaction processes I: Finite element solutions. Journal of Computational Physics. 2010;229(19):6979–6994. 10.1016/j.jcp.2010.05.035 21709855 PMC2922884

[120] Alnæs MS, Blechta J, Hake J, Johansson A, Kehlet B, Logg A, et al The FEniCS Project Version 1.5. Archive of Numerical Software. 2015;3(100).

