# Supplementary material for: Co-localization and confinement of ecto-nucleotidases modulate extracellular adenosine nucleotide distributions
Source: PLoS Comput Biol. 2020 Jun 25;16(6):e1007903. doi: 10.1371/journal.pcbi.1007903 (PMC7316229; doi:10.1371/journal.pcbi.1007903)
Supplement: S1 Figures — (PDF) [file pcbi.1007903.s001.pdf]

S.1 Figures

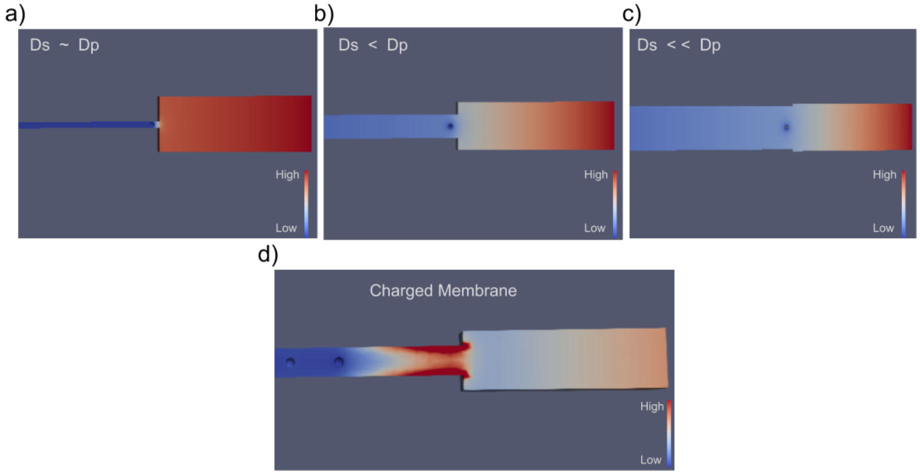

Fig A. ATP concentration profiles. Panels a)-c) represent uncharged systems with only a CD39 enzyme, in order of increasing junction radius, whereas d) is a solution for $z_{ATP} = -2$ and $\Phi_{junction} > 0$ and both CD39 and CD73 enzymes. The inset circles are the spherical inclusions representing the CD39 and/or CD73 enzymes. The spheres have a Dirichlet boundary condition of zero ATP concentration on their surface, resulting in a blue color in these images .

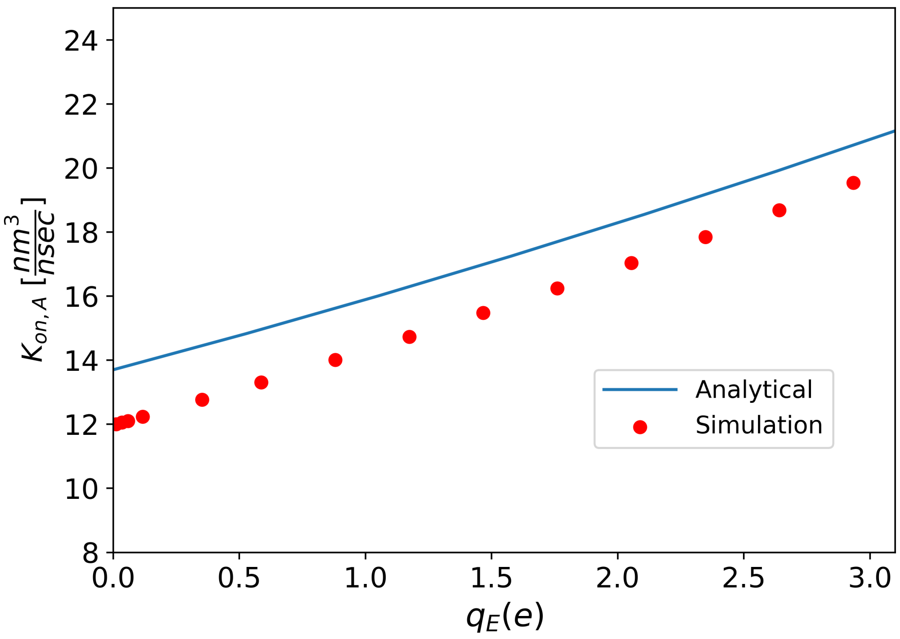

Fig B. Validation of solution to the electro-diffusion equation for a single enzyme under bulk-like conditions. $k_{on,ATP}$ obtained from theoretical prediction (see Eq. 3 in Sect.) and simulation data for a bulk-like system with a single CD39 enzyme .

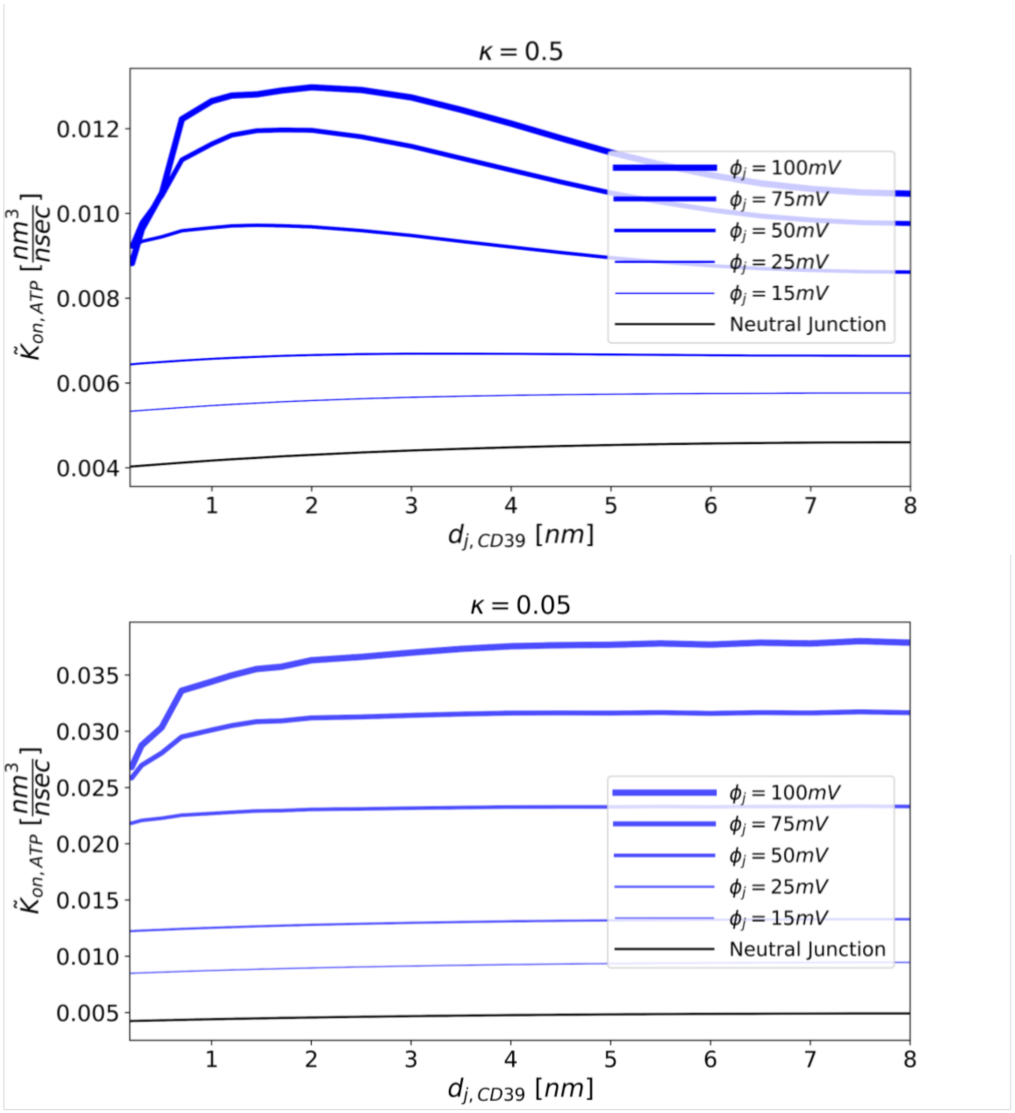

Fig C. Predictions of $k_{on,ATP}$ as a function of junction/CD39 distance (d_{tether}), membrane potential and ionic strength (κ). Note that for simplicity we assumed the linearized Poisson-Boltzmann equation here, thus the quantitative accuracy of the potentials > 25 mV is limited .

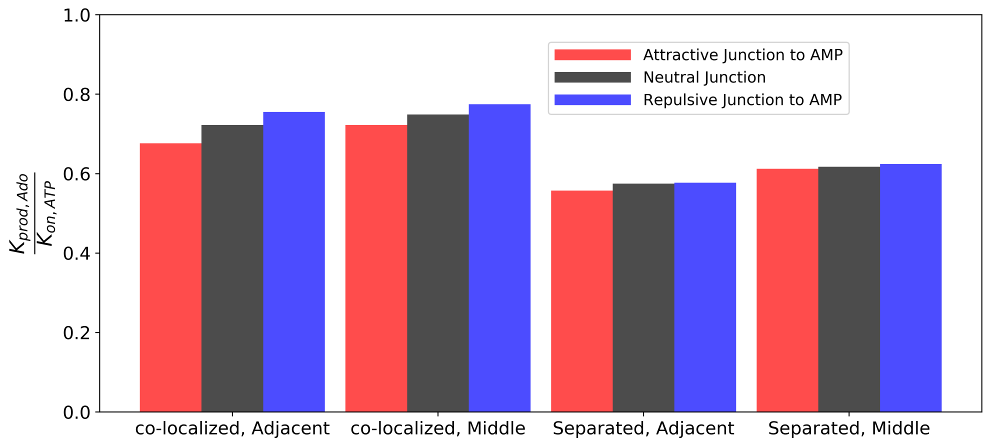

Fig D. Efficiency of AMP to Ado conversion via CD39 and CD73 catabolism. k_{eff} values are reported for attractive versus repulsive junctional membrane potentials, degree of CD39/CD73 co-localization, and adjacency of the junctional membrane surface. All calculations assume z_{ATP}=-2, z_{AMP}=-1, z_{Ado}=0 and $\Phi_{CD39} > 0$. .
