# Supplementary material for: Co-localization and confinement of ecto-nucleotidases modulate extracellular adenosine nucleotide distributions
Source: PLoS Comput Biol. 2020 Jun 25;16(6):e1007903. doi: 10.1371/journal.pcbi.1007903 (PMC7316229; doi:10.1371/journal.pcbi.1007903)
Supplement: S1 Text — With supporting figures. (PDF) [file pcbi.1007903.s002.pdf]

S.1 Text

Electrostatic analogy to diffusion problems

The Fickian diffusion model is mathematically similar to the Laplace equation of electrostatics. This similarity can be exploited to allow techniques developed for electrostatics problems to be applied to Fickian diffusion problems as well.

Starting with similarity of Laplace equation in electrostatics and diffusion equation in steady state condition:

$$\nabla^2 \Phi = 0 \tag{S1}$$

$$D\nabla^2 c = 0 \tag{S2}$$

This similarity implies an equivalency between electrostatic parameters and diffusion ones:

$$\Phi \to Dc \tag{S3}$$

$$E \to J \tag{S4}$$

Interestingly, since $q_{in} = \int_\Gamma \vec{E} \cdot \hat{n} d\Gamma$ and according to what we defined for reactivity $k_{on} = \int_\Gamma \vec{J} \cdot \hat{n} d\Gamma$, we can consider:

$$q_{in} \to k_{on} \tag{S5}$$

One interesting relation that we can use is coming from the relation between capacitance and total charge on a conductor in electrostatics:

$$q = 4\pi C\phi \tag{S6}$$

where C is the capacitance of the conductor and ϕ is the electrical potential. By using the similarity between q and k we can have:

$$k_{on} = 4\pi CD \tag{S7}$$

For example, a spherical capacitor with inner radius of R and outer radius of r has a capacitance of $rR/(r-R)$, so that:

$$k_{on} = 4\pi RD \frac{1}{r/R - 1} \tag{S8}$$

which is the same as Eq. 2. In a spacial case in which outer radius is infinity (a single spherical conductor in the bulk), we have C=R, which leading to:

$$k_{on} = 4\pi RD \tag{S9}$$

These equations demonstrate the analogy of the charge, q, contained with the surface and reaction rates. For instance, we can also use Eq. S7 to validate confinement effects on reaction rate in Sect. . In the electrostatic analog, the sphere has a surface charge and the junction is another conductor with the same charge sign, with the reservoir a source with opposite charge. Since the total electric flux, charge, is dependent on the capacitance, as the charged sphere approaches a wall ($J \cdot n = 0$), the capacitance must decrease [1] and thereby reduce the total electric flux. By analogy, the numerically-estimated $k_{on,ATP}$ values were smaller for immobilized enzymes relative to those far from the surface. We also applied this similarity to sequential enzymes in the following section.

Comparison to the method of image charges

Here we consider the two enzymes equivalent to the boundary value problem for describing the electric potential for a system comprising two conductive spheres with different electric potentials (see Fig E).

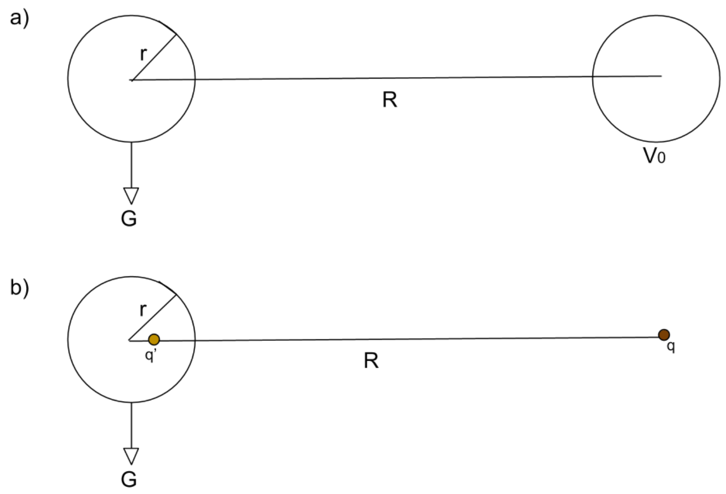

Fig E. Conductive spheres physical geometry. a)Physical geometry of two conductive spheres similar to the sequential enzymes. b)Simplified geometry considering one of the spheres as a point charge to obtain electrical potential near the remaining sphere. .

For simplicity, we will calculate the electrical potential in vicinity of the second sphere, by assuming that the first enzyme is a point charge, q, which is proportional to its potential, ϕ_0:

$$q = r\phi_0 \tag{S10}$$

To obtain the electrical potential we applied ”method of image charges”, in which we consider imaginary charges to replicate boundary conditions of the system. Here, to obtain the electrical potential outside the second enzyme, we must put some image charge inside it, considering its surface as grounded (zero) potential, as the surface of the second enzyme is completely absorbing (c=0). This boundary condition can be satisfied by putting an image charge q' proportional to $\frac{1}{R}$, on r' from the second sphere and on the line connecting both spheres, which should be placed in $\frac{r^2}{R}$([2], chapter 2):

$$q' = \frac{r^2}{R}\phi_0 \tag{S11}$$

$$r' = \frac{r^2}{R} \tag{S12}$$

Therefore, the total charge inside the second sphere is proportional to the inverse of the distance from the first enzyme. The equivalency of total charge and reaction rate of the second enzyme equivalate the same dependancy of k_{on} to the distance between sequential enzymes:

$$k_{on,R} = \frac{k_{on,colocalized}}{R} \tag{S13}$$

To examine this fact on our simulation, we compared the simulation result with what we predicted from the analytical approach, as shown Fig F:

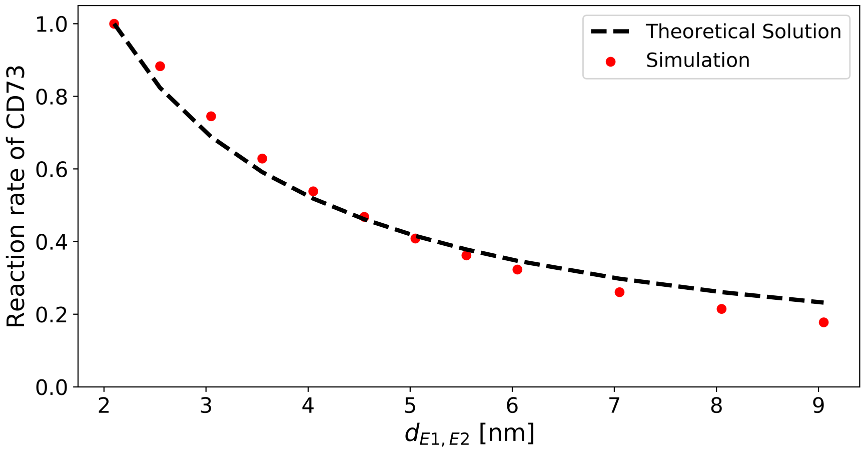

Fig F. Validation of sequential enzymes' simulation. Validation of sequential enzymes simulation in bulk-like media by comparison to predictions (see Eq. S13) obtained from an analytical approach by analogy to the ”method of image charges” in electrostatics. The data for both series are normalized to their own result for the smallest enzyme separation distance .

We used this equivalency to validate our sequential enzymes model simulation.

Reactivity of a charged single enzyme in bulk based on electrostatic interaction energy

In Figure B, we compare the analytical solution from Eq. 3 to the results of finite element simulations under bulk-like conditions with a single CD39 enzyme. The two curves are approximately similar. The difference between the two curves is likely due to the the differing assumptions. Eq. 3 is derived under an assumption of perfect spherical symmetry, which is not present in the junction model. Furthermore, Eq. 3 assumes $\kappa \to 0$, whereas the simulations were conducted with a finite value of $\kappa = 0.05$.

Effects of enzyme and junctional membrane charge on $k_{prod,Ado}$ and k_{eff}

We found in Sect. that attractive interactions between substrate ATP and a positively-charged surface lead to the greatest overall enhancements of $k_{prod,Ado}$. There is, however, a limit to this acceleration, if the attractive interactions between the junction and substrate are stronger than the target enzyme, as we demonstrate with a reduction in $k_{on,ATP}$ demonstrated in Fig C. To assess the extent to which AMP/junction interactions contribute to $k_{prod,Ado}$, we examined the k_{eff} ratios for the nonreactive junction boundaries. Based on the efficiencies reported in Fig D, most of the increased production rate can be attributed to $k_{on,ATP}$, as the efficiencies were largely constant across the various charge configurations. However, efficiencies were generally greater for co-localized enzymes versus separated configurations, regardless of the junction charge. For negatively-charged junctions ($\phi_{junction} < 0$), the reaction efficiency was enhanced for all configurations. The increased efficiency for negatively-charged junctions can be rationalized by the pushing of anionic AMP toward vicinity of enzymes, However due to considerably more favorable effect of attractive junction on ATP, in overal, attractive junction increases $k_{on,AMP}$. This concurs with our findings of enhanced reaction efficiency in DHFR-TS [3] owing to its complementary surface charge to the dihydrofolate intermediate intermediate, in contrast to a neutral or electrically-repulsive surface. For the co-localized cases at the membrane surface, it is likely that the membrane significantly competed with the binding of AMP at CD73, which led to a reduced reaction rate coefficient. Overall, these results suggest that favorable ATP/membrane interactions largely control the absolute $k_{prod,Ado}$ rate, with co-localization typically further enhancing $k_{prod,Ado}$and k_{eff}. The contributions of AMP/junction interactions play a comparatively smaller role, perhaps given the limited extent to which AMP permeated the junction relative to ATP.

References

1. Berg HC, Purcell EM. Physics of chemoreception. Biophysical Journal. 1977;20(2):193–219.

2. Jackson JL, Coriell SR. Transport Coefficients of Composite Materials. Journal of Applied Physics. 1968;39(5):2349–2354. doi:10.1063/1.1656558.

3. Metzger VT, Eun C, Kekenes-Huskey PM, Huber G, McCammon JA. Electrostatic channeling in P. falciparum DHFR-TS: Brownian dynamics and smoluchowski modeling. Biophys J. 2014;107(10):2394–2402. doi:10.1016/j.bpj.2014.09.039.
