# Supplementary material for: Co-localization and confinement of ecto-nucleotidases modulate extracellular adenosine nucleotide distributions
Source: PLoS Comput Biol. 2020 Jun 25;16(6):e1007903. doi: 10.1371/journal.pcbi.1007903 (PMC7316229; doi:10.1371/journal.pcbi.1007903)
Supplement: S2 Text — (PDF) [file pcbi.1007903.s003.pdf]

S.2 Text

Mathematical Notation

Φ_{CD39} Electric potential on the surface of CD39.

Φ_{CD73} Electric potential on the surface of CD73.

$\Phi_{junction}$ Electric potential on the boundary surface of the junction.

Γ_{CD39} Boundary surface of the model at CD39.

Γ_{CD73} Boundary surface of the model at CD73.

Γ_j Boundary surface of the model at the junction.

Γ_L Boundary surface of the model at the left reservoir.

Γ_R Boundary surface of the model at the right reservoir.

$d_{CD39,CD73}$ Center-to-center separation distance between CD39 and CD73.

d_{tether} Distance from center of enzyme to nearest point on junction wall.

k_{eff} Ratio of Ado production at CD73 to rate of ATP association at CD39.

$k_{on,ATP}$ Rate of ATP association at CD39.

$k_{on,AMP}$ Rate of AMP association at CD73.

$k_{prod,AMP}$ Rate of AMP production at CD39.

$k_{prod,Ado}$ Rate of Ado production at CD73.

r_E Radius of sphere representing CD39 or CD73 enzyme.

r_j Radius of cylindrical junction in the model.

Abbreviations

Ado adenosine.

ADP adenosine diphosphate.

AMP adenosine monophosphate.

ATP adenosine triphosphate.

DHFR-TS dihydrofolate reductase-thymidylate synthase.

NDA ecto-nucleotidase.

ODE ordinary differential equation.
